# Rampant Misexpression in a *Mimulus* (Monkeyflower) Introgression Line Caused by Hybrid Sterility, Not Regulatory Divergence

**DOI:** 10.1093/molbev/msaa071

**Published:** 2020-03-20

**Authors:** Rachel E Kerwin, Andrea L Sweigart

**Affiliations:** m1 Department of Genetics, University of Georgia, Athens, GA; m2 Department of Biochemistry and Molecular Biology, Michigan State University, East Lansing, MI

**Keywords:** monkeyflower, *Mimulus*, allele-specific expression, regulatory divergence, Dobzhansky–Muller incompatibility, hybrid male sterility

## Abstract

Divergence in gene expression regulation is common between closely related species and may give rise to incompatibilities in their hybrid progeny. In this study, we investigated the relationship between regulatory evolution within species and reproductive isolation between species. We focused on a well-studied case of hybrid sterility between two closely related yellow monkeyflower species, *Mimulus guttatus* and *Mimulus nasutus*, that is caused by two epistatic loci, *hybrid male sterility 1* (*hms1*) and *hybrid male sterility 2* (*hms2*). We compared genome-wide transcript abundance across male and female reproductive tissues (i.e., stamens and carpels) from four genotypes: *M. guttatus, M. nasutus*, and sterile and fertile progeny from an advanced *M. nasutus*–*M. guttatus* introgression line carrying the *hms1–hms2* incompatibility. We observed substantial variation in transcript abundance between *M. guttatus* and *M. nasutus*, including distinct but overlapping patterns of tissue-biased expression, providing evidence for regulatory divergence between these species. We also found rampant genome-wide misexpression, but only in the affected tissues (i.e., stamens) of sterile introgression hybrids carrying incompatible alleles at *hms1* and *hms2*. Examining patterns of allele-specific expression in sterile and fertile introgression hybrids, we found evidence for interspecific divergence in *cis-* and *trans-*regulation, including compensatory *cis–trans* mutations likely to be driven by stabilizing selection. Nevertheless, species divergence in gene regulatory networks cannot explain the vast majority of the gene misexpression we observe in *Mimulus* introgression hybrids, which instead likely manifests as a downstream consequence of sterility itself.

## Introduction

Closely related species often show considerable regulatory divergence—that is, they have accumulated differences in the *cis*-acting DNA sequences or *trans*-acting factors that regulate gene expression (Tautz 2000; Wray et al. 2003). As with any epistatic loci, divergence in interacting regulatory elements might lead to incompatibilities in the hybrid progeny of interspecific crosses (Dobzhansky 1937; Muller 1942; Mack and Nachman 2017). Such hybrid incompatibilities might arise due to independent substitutions in distinct lineages, with genetic drift or selection increasing the frequency of a derived allele at a *cis*-acting locus in one species and a *trans*-acting partner locus in the other. In the classic Dobzhansky–Muller model, these derived alleles are neutral or favored on their own but cause aberrant gene expression when combined in hybrids. Alternatively, hybrid incompatibilities might arise because of coevolution between *cis*- and *trans*-elements within a single lineage. Stabilizing selection to maintain optimal levels of transcription, which favors *cis*- and *trans*-regulatory variants that compensate for each other, appears to be an important force shaping gene expression evolution (Gilad et al. 2006; Tirosh et al. 2009; Goncalves et al. 2012; Coolon et al. 2014; Mack et al. 2016). Thus, even when transcript abundance for a particular gene does not differ between species, the underlying regulatory components controlling its expression might have diverged (Tautz 2000; True and Haag 2001; Wray et al. 2003). This process of compensatory evolution in gene regulatory networks, which is likely to affect different sets of genes in diverging lineages, has been proposed as a major source of hybrid incompatibilities between species (Landry et al. 2005; Takahasi et al. 2011).

Despite the clear importance of changes in gene expression for phenotypic evolution (Wittkopp 2013), empirical support for regulatory divergence as a general driver of hybrid incompatibilities is mixed. Although many studies have uncovered pervasive gene misexpression in sterile hybrids (i.e., over- or under-expression in hybrids relative to both parental species: Michalak and Noor 2003; Ranz et al. 2004; Haerty and Singh 2006; Malone et al. 2007; Coolon et al. 2014; Brill et al. 2016; Mack et al. 2016), others have found no association between patterns of gene expression and hybrid dysfunction (Barbash and Lorigan 2007; Wei et al. 2014; Guerrero et al. 2016). Even when sterile or inviable hybrids *do* show widespread misexpression, it does not necessarily imply that regulatory divergence between species is the cause. Rather, genome-wide misexpression might occur as a downstream consequence of one or a small number of hybrid incompatibilities that effect gene regulation. It could also arise as a byproduct of hybrid dysfunction itself, which often involves gross defects in affected tissues (e.g., testes in male sterile hybrids) and abnormal or missing cell types. For example, although misexpression increases dramatically in hybrids between *Drosophila* species pairs with longer divergence times (Coolon et al. 2014), so does the severity of hybrid dysfunction.

A promising approach to disentangle the causes of hybrid incompatibilities from their downstream effects is to examine interspecific gene expression variation associated with particular genomic regions. Although most studies of regulatory divergence compare gene expression profiles between parental species and F_1_ hybrids, a handful have used introgression lines (Lemos et al. 2008; Meiklejohn et al. 2014; Guerrero et al. 2016) or recombinant mapping populations (Turner et al. 2014), which can facilitate investigations into whether gene regulation and hybrid dysfunction have a shared genetic basis. With the introgression approach, it is possible to examine the regulatory effects of small genomic segments from one species on the genetic background of another species. If regulatory incompatibilities (i.e., gene misexpression in hybrids due to divergence in interacting *cis*- and *trans*-factors) are a common outcome of species divergence in gene regulatory networks, introgression of nearly any heterospecific segment is likely to cause some degree of gene misexpression. On the other hand, if widespread misexpression is confined to introgression hybrids carrying known incompatibility alleles, it is likely to be a downstream effect of hybrid dysfunction.

**Fig. 1.**
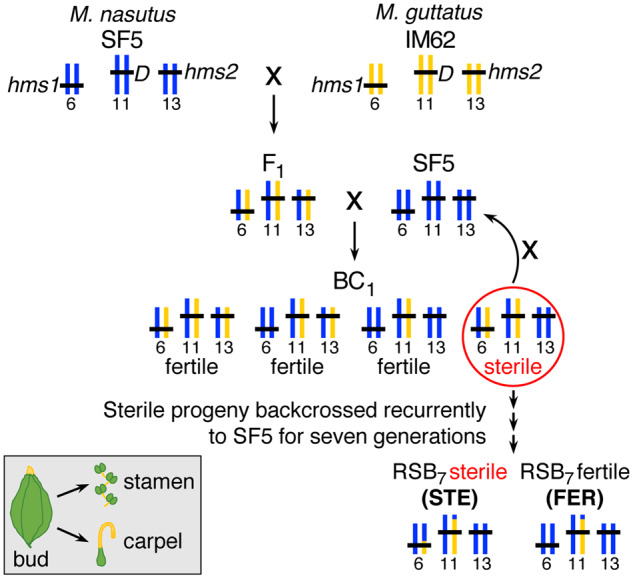
Crossing scheme to generate RSB introgression hybrids. First, *Mimulus nasutus* SF5 and *Mimulus guttatus* IM62 were cross pollinated, yielding an SF5–IM62 F_1_ that was subsequently backcrossed to SF5, yielding a first-generation backcross (BC_1_) population. A pollen sterile individual from the BC_1_ population (circled in red) was selected and backcrossed to SF5, yielding a first-generation RSB introgression line (RSB_1_). This selective backcrossing was repeated for six more generations, producing an RSB_7_ population. Roughly, 50% of the RSB_7_ siblings are pollen STE because they carry an heterozygous introgression on chromosome 6 (with an incompatible IM62 allele at *hms1*) in an SF5 genomic background (that is fixed for the incompatible SF5 allele at *hms2*), whereas the other 50% are pollen FER because they carry only SF5 alleles at *hms1* (in the same genomic background that is fixed for the incompatible SF5 allele at *hms2*). Whole transcriptome sequencing was performed on RNA extracted from three biological replicates each of stamens and carpels (gray box, bottom left) from four genotypes, *M. nasutus* SF5, *M. guttatus* IM62, FER RSB_7_ introgression hybrid, and STE RSB_7_ introgression hybrid, for a total of 24 samples, representing eight tissue-genotype categories. To obtain sufficient RNA for sequence library preparation, we dissected four to eight floral buds each biological replicate. *D* = meiotic drive locus (Fishman and Willis 2005).

To investigate the link between regulatory divergence and hybrid dysfunction, we exploited a well-studied hybrid incompatibility system between two closely related species of monkeyflower (*Mimulus*). Previously, we discovered severe male sterility and partial female sterility in hybrids between *Mimulus guttatus* (IM62 inbred line) and *Mimulus nasutus* (SF5 inbred line) (Sweigart et al. 2006) and fine mapped the effects to two small nuclear genomic regions of ∼60 kb each on chromosomes 6 and 13 (Sweigart and Flagel 2015). Hybrids that carry at least one incompatible *M. guttatus* IM62 allele at *hybrid male sterility 1* (*hms1*) on chromosome 6 in combination with two incompatible *M. nasutus* SF5 alleles at *hybrid male sterility 2* (*hms2*) on chromosome 13 display nearly complete (>90%) pollen sterility, whereas other allelic combinations are highly fertile (Sweigart et al. 2006; Sweigart and Flagel 2015; Kerwin and Sweigart 2017). Here, we took advantage of SF5–IM62 introgression hybrids, formed through multiple rounds of selection for pollen sterility and backcrossing (as the female parent) to *M. nasutus* SF5 (fig. 1). This *r*ecurrent *s*election with *b*ackcrossing (RSB) population maintains a heterozygous IM62 introgression on chromosome 6 (that contains the *hms1* locus) against a primarily SF5 genetic background (including at *hms2*). Each generation, RSB progeny segregate ∼1:1 for sterility to fertility, depending on whether they inherit a copy of the incompatible IM62 *hms1* allele. Additionally, nearly all RSB hybrids are expected to carry a non-sterility-causing, heterozygous IM62 introgression on chromosome 11 surrounding the female meiotic drive locus (*D*), which is transmitted to >98% of progeny when an SF5–IM62 F_1_ hybrid acts as the maternal parent (Fishman and Willis 2005). Thus, this crossing scheme produces two classes of progeny: sterile (STE) individuals that carry two heterozygous introgressions (on chromosome 6 with *hms1* and on chromosome 11 with *D*) and fertile (FER) individuals that carry a single introgression (on chromosome 11 with *D*). The result is an internally controlled genetic experiment that is ideally suited for determining whether gene misexpression is a cause or consequence of hybrid sterility.

For this study, we sequenced the transcriptomes of FER and STE individuals from a seventh-generation RSB population (RSB_7_) alongside their parents, *M. guttatus* IM62 and *M. nasutus* SF5. Because the *hms1–hms2* incompatibility affects both male and female fertility (Sweigart et al. 2006), we isolated RNA separately from developing stamens and carpels. This RNAseq data set allowed us to identify changes in gene expression due to the presence of a genomic segment that carries a known hybrid sterility allele (i.e., the IM62 allele at *hms1*) versus a genomic segment that does not (i.e., the IM62 allele at *D*). If regulatory incompatibilities between *Mimulus* species are common, we would expect to see misexpression (i.e., expression that falls outside the parental range) in both FER and STE introgression hybrids, due to heterospecific combinations of divergent *cis*- and *trans*-regulatory alleles. If instead, misexpression is confined to the affected tissues (i.e., stamens) of the STE introgression hybrid, it might suggest that downstream effects of the *hms1–hms2* incompatibility are causal. With this RNAseq data set, we addressed the following specific questions. To what extent does gene expression vary between closely related *Mimulus* species? Are patterns of tissue-biased expression in stamens and carpels conserved between species? What are the relative contributions of *cis-* and *trans-*regulatory divergence to interspecific variation in gene expression? Is there an association between misexpression and hybrid sterility and, if so, what is its cause? Do expression patterns narrow down the list of candidate genes for *hms1* or *hms2*? Our results provide insight into regulatory divergence between closely related species and the consequences for reproductive isolation.

## Results

To examine patterns of genome-wide expression associated with the *hms1–hms2* hybrid incompatibility in *Mimulus*, we performed transcriptome sequencing (RNAseq) on stamens and carpels from *M. guttatus* IM62, *M. nasutus* SF5, and FER and STE progeny of an advanced SF5–IM62 introgression population called *r*ecurrent *s*election with *b*ackcrossing (RSB_7_) (fig. 1). We obtained an average of 14.1 million (range, 10.9–16.8 million) 50-bp single-end reads from each sample (supplementary table S1, Supplementary Material online). After initial processing, we aligned trimmed reads to a diploid *M. guttatus* IM62–*M. nasutus* SF5 pseudoreference genome generated for this study (see Materials and Methods for details). An average of 44.6% (range, 35.6–49.5%) of the reads were unambiguously assigned to an IM62 or SF5 allele (i.e., allele specific). An additional 34% (range, 29.5–36.9%) of the reads mapped to a single genomic location but could not be assigned to an IM62 or SF5 allele, due to an absence of distinguishing single-nucleotide polymorphisms (SNPs) (supplementary table S1, Supplementary Material online). Across the 14 chromosomes, 20,431 of 28,140 predicted genes were expressed (i.e., read count-per-million [CPM] >1 in >2 samples).

To determine introgression boundaries in the STE and FER RSB_7_ samples, we assigned genotypes using allele-specific reads (see Materials and Methods for details). As expected, STE and FER genotypes differ only in the region surrounding *hms1* on chromosome 6: STE individuals contain a heterozygous IM62 introgression that stretches across a 7-Mb region encoding 699 genes (498 of which are expressed in our data set), whereas FER individuals are homozygous for the recurrent SF5 parent (supplementary fig. S1, Supplementary Material online). Additionally, both FER and STE individuals carry a large heterozygous IM62 introgression that spans 23 Mb of chromosome 11 (90% of the physical distance; supplementary fig. S1, Supplementary Material online), a region that encodes 1,066 genes (747 expressed in our data set) and harbors a female meiotic drive locus (*D*) associated with strong transmission ratio distortion in SF5–IM62 hybrids (Fishman and Willis 2005; Fishman and Saunders 2008).

### Expression Variation Is Driven by Species, Tissue, and Fertility

**Fig. 2.**
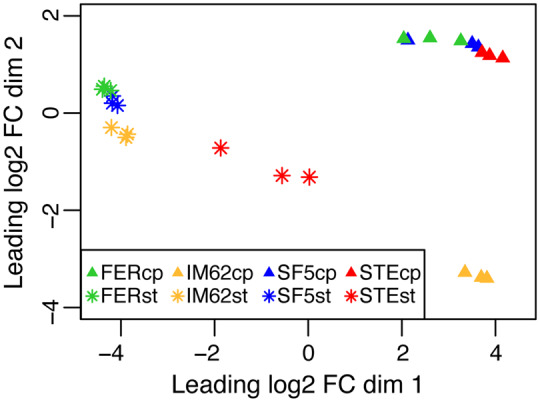
Genome-wide expression pattern across samples. Shown is a MDS plot comparing gene expression across the 24 RNAseq samples in our data set. Distance between points represents the leading (i.e., largest absolute) log 2 fold-change (FC) transcript abundance between pairs of samples mapped onto two-dimensional space: the *y*-axis shows dimension 1 (dim 1) and the *x*-axis shows dimension two (dim 2). The colors and shapes represent the different genotype-tissue categories. IM62, *Mimulus guttatus* IM62 parent; SF5, *Mimulus nasutus* SF5 parent; FER, fertile RSB_7_ introgression hybrid; STE, sterile RSB_7_ introgression hybrid; FERcp, FER carpel; FERst, FER stamen; IM62cp, IM62 carpel; IM62st, IM62 stamen; SF5cp, SF5 carpel; SF5st, SF5 stamen; STEcp, STE carpel; STEst, STE stamen.

To visualize genome-wide expression patterns across the 24 samples in our data set, we generated a multidimensional scaling (MDS) plot (fig. 2). Replicate samples from each of the eight genotype-tissue groups form clusters that are generally separated from other groups (fig. 2). Along the *x*-axis of the MDS plot, expression variation is largely determined by tissue type with clear separation between carpel and stamen samples (fig. 2). Along the *y*-axis, samples are differentiated by a combination of tissue type and species identity, with stamens and carpels clustering separately, and IM62 clustering separately from SF5, FER, and STE (the latter two carrying SF5 variation across most of their genetic backgrounds; see supplementary fig. S1, Supplementary Material online).

**Fig. 3.**
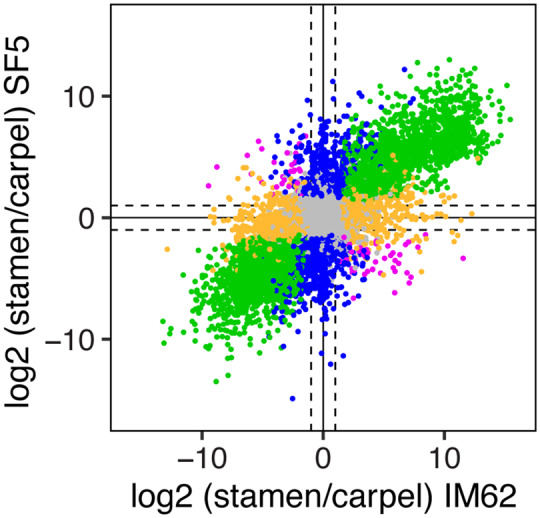
Parental tissue-biased gene expression. Scatterplot shows relative transcript abundance (log 2 fold-change) between stamen and carpel tissues in the SF5 and IM62 parents. Of the 20,431 genes expressed in our data set, 1,812 (8.9%) and 5,700 (27.9%) were significantly (log 2 FC > 1.25, FDR ≤ 0.05) stamen biased (green points in top right quadrant) and carpel biased (green points in bottom left quadrant), respectively, in both parents. An additional 546 (2.7%) and 1,339 (6.6%) genes were stamen biased and carpel biased, respectively, in SF5 only (blue points), whereas 615 (3%) and 2,228 (10.9%) genes were stamen biased and carpel biased, respectively, in IM62 only (yellow points). A few genes exhibited opposing tissue-biased expression patterns in SF5 and IM62 (purple points): 28 (0.1%) were stamen biased in SF5 and carpel biased in IM62, whereas 50 (0.2%) were carpel biased in SF5 and stamen biased in IM62. The remaining 8,113 (39.7%) genes were evenly expressed between parental tissues (gray points). IM62, *Mimulus guttatus* IM62 parent; SF5, *Mimulus nasutus* SF5 parent.

To investigate these differences more thoroughly, we compared patterns of tissue-biased gene expression between IM62 and SF5 (fig. 3). For a large number of genes, tissue-biased expression is conserved between species: 7,512 genes (37%) show higher or lower expression in the same tissues in both IM62 and SF5 (green points in fig. 3). On the other hand, a considerable number of genes (4,728, 23%; blue and yellow points in fig. 3) show tissue-biased expression in only one of the two species and a handful (78, 0.4%; purple points in fig. 3) even show opposite patterns of tissue-biased expression. We also looked more closely at interspecific expression variation between IM62 and SF5 within carpels and stamens (supplementary fig. S2, Supplementary Material online). Among the 20,431 expressed genes in our data set, we found that 2,037 (10%) and 2,341 (11%) were significantly differentially expressed (log 2 fold-change > 1.25, false discovery rate [FDR] ≤ 0.05) between SF5 and IM62 in carpels and stamens, respectively (supplementary fig. S2, Supplementary Material online).

**Fig. 4.**
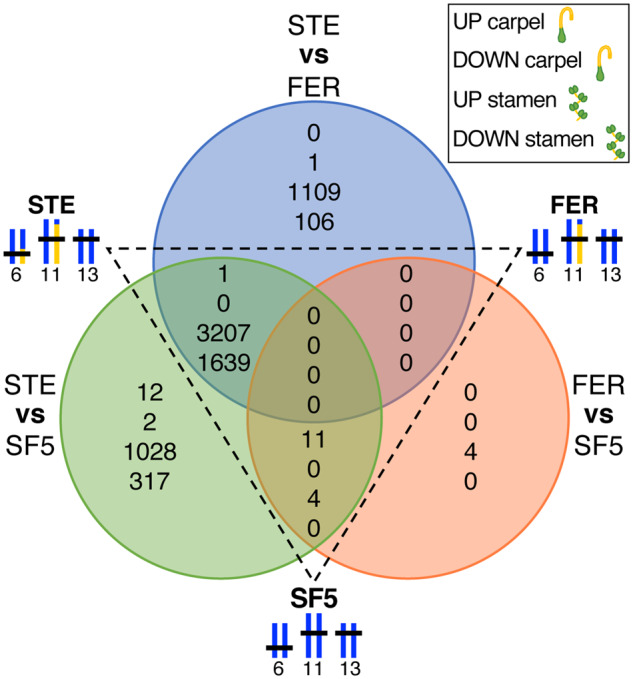
Gene expression differences across genotypes and tissues. Venn diagrams show counts of genes with significantly altered transcript abundance (log 2 fold-change > 1.25, FDR ≤ 0.05) in carpels and stamens across three pairwise comparisons: 1) STE versus FER, 2) STE versus SF5, and 3) FER versus SF5. SF5, *Mimulus nasutus* SF5 parent; FER, fertile RSB_7_ introgression hybrid; STE, sterile RSB_7_ introgression hybrid.

A conspicuous exception to the species-tissue clustering pattern just described is the distinct group formed by the STE stamen samples (see red asterisks in fig. 2). We reasoned that this unique pattern of gene expression in STE stamens might be driven by the presence of the chromosome 6 introgression, which carries the hybrid male sterility-causing IM62 *hms1* allele. To investigate this possibility, we compared gene expression profiles among STE, FER, and SF5 samples (fig. 4). Indeed, the vast majority of differentially expressed genes were identified in comparisons of stamens with and without the chromosome 6 introgression: of the 20,431 genes expressed in our data set, 4,846 (24%) were differentially expressed (3,207 upregulated, 1,639 downregulated) in stamens across both STE versus FER and STE versus SF5 (fig. 4). Moreover, differentially expressed STE stamen genes were distributed throughout the genome, with roughly equal proportions in the introgression (25%, 313/1,245) and background (24%, 4,512/19,186) regions (supplementary fig. S3, Supplementary Material online). In contrast, far fewer expression differences were found in comparisons of stamens distinguished only by the chromosome 11 introgression (fig. 4, FER vs. SF5 and STE vs. SF5: 4 upregulated, 0 downregulated). Additionally, relatively few genes were differentially expressed in carpels, whether they carried the chromosome 6 introgression or not (across all comparisons: 24 upregulated, 3 downregulated). Despite the fact that the chromosome 6 introgression causes a substantial reduction in female fertility in STE hybrids (supplemental seed set of FER RSB_3_ is >4× that of STE RSB_3_, fig. 6 in Sweigart et al. 2006), it appears to have little effect on gene expression in the carpels we collected.

### Rampant Misexpression Is Confined to Tissues Affected by Hybrid Male Sterility

**Fig. 5.**
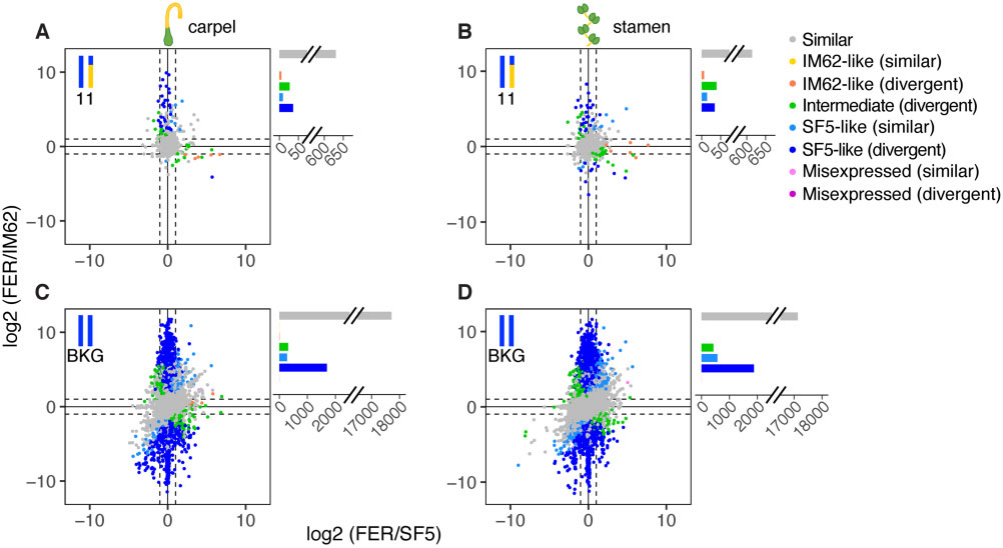
Genome-wide pattern of expression in FER introgression hybrids. Points represent relative transcript abundance (log 2 fold-change) between FER and SF5 on the *x*-axis and between FER and IM62 on the *y*-axis across (*A*, *C*) carpels and (*B*, *D*) stamens for (*A*, *B*) heterozygous genes in the chromosome 11 introgression and (*C*, *D*) homozygous background genes. Points are colored by expression class (see supplementary table S2, Supplementary Material online, for description). IM62, *Mimulus guttatus* IM62 parent; SF5, *Mimulus nasutus* SF5 parent; FER, fertile RSB_7_ introgression hybrid.

**Fig. 6.**
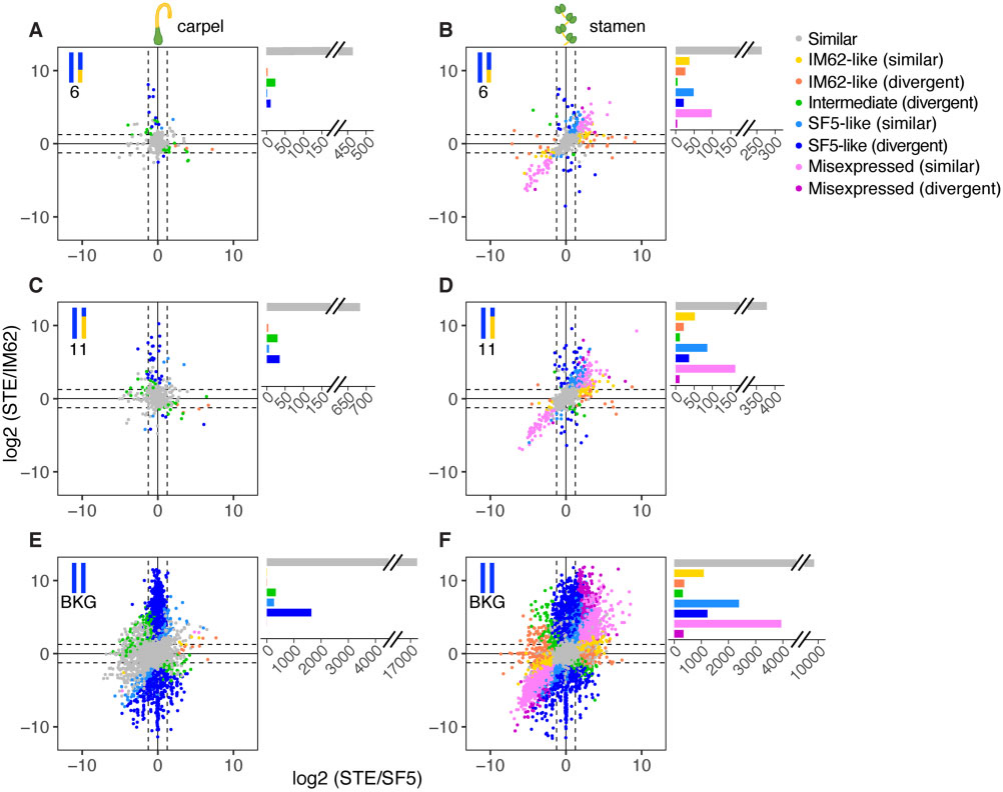
Genome-wide pattern of expression in STE introgression hybrids. Points represent relative transcript abundance (log 2 fold-change) between STE and SF5 on the *x*-axis and between STE and IM62 on the *y*-axis across (*A*, *C*, *E*) carpels and (*B*, *D*, *F*) stamens for heterozygous genes in the (*A B*) chromosome 6 and (*C*, *D*) chromosome 11 introgressions, and (*E*, *F*) homozygous background genes. Points are colored by expression class (see supplementary table S2, Supplementary Material online, for description). IM62, *Mimulus guttatus* IM62 parent; SF5, *Mimulus nasutus* SF5 parent; STE, sterile RSB_7_ introgression hybrid.

To further examine the effects of the chromosome 6 and chromosome 11 introgressions on transcriptional regulation, we characterized gene expression in FER (fig. 5) and STE (fig. 6) tissues relative to both parents (supplementary table S2, Supplementary Material online). Because patterns of relative expression are expected to differ between heterozygous genes located within the introgressions and homozygous genes located in the background (which carry two SF5 alleles), we plotted these gene classes separately.

Patterns of relative transcript abundance in FER carpels, FER stamens, and STE carpels (i.e., tissues unaffected by hybrid sterility) are reflective of both additive and dominant inheritance of regulatory variants from SF5 and IM62. Among the heterozygous introgression genes that exhibited significant (log 2 fold-change > 1.25, FDR ≤ 0.05) parental expression divergence (labeled “divergent” in figs. 5 and 6), up to half showed intermediate levels of expression in FER carpels (39% [26/66]), FER stamens (50% [39/78]), and STE carpels (51% [53/104]) (green points/bars in figs. 5*A* and *B* and 6*A* and *C* and supplementary table S3, Supplementary Material online), consistent with the additive effects of *cis*-regulatory alleles. The other half of these divergent heterozygous introgression genes exhibited SF5-like or IM62-like expression (dark blue and orange points/bars in figs. 5*A* and *B* and 6*A* and *C* and supplementary table S3, Supplementary Material online), which could result from dominance of one *cis*-acting variant over the other. Consistent with this possibility, expression of heterozygous genes usually matched the more highly expressed parent (note the bias toward positive values in figs. 5*A* and *B* and 6*A* and *C*). Dominance of a high-expression allele might be caused by a *cis*-mutation that increases its own transcription (whereas a dominant *cis*-acting, low-expression allele would have to *interfere* with expression from the alternate allele). Dominant effects of *trans*-acting factors on gene expression in the heterozygous introgressions are also apparent: More divergent genes exhibited SF5-like than IM62-like expression, suggesting a strong influence of *trans*-factors from the SF5 background (compare dark blue SF5-like points/bars with orange IM62-like bars in figs. 5*A* and *B* and 6*A* and *C* and supplementary table S3, Supplementary Material online). Similarly, although 82–85% of the divergent background genes were SF5-like due to their homozygosity for SF5 alleles (blue points/bars in figs. 5*C* and *D* and 6*E* and supplementary table S3, Supplementary Material online), a substantial proportion (15–18%) showed intermediate expression (green points/bars) signifying incomplete dominance of IM62 *trans*-alleles from the heterozygous introgressions. Very few divergent background genes were IM62-like (orange points/bars) or misexpressed (light and dark pink points/bars; figs. 5*C* and *D* and 6*E* and supplementary table S3, Supplementary Material online).

The pattern of expression in STE stamens differed dramatically from all other samples, with a large number of genes misexpressed (22% [4,533/20,431]; light and dark pink points/bars in fig. 6*B*, *D*, and *F* and supplementary table S3, Supplementary Material online). The vast majority of misexpression was found in genes that exhibited expression conservation between the parents (light pink points/bars in fig. 6*F*). Additionally, STE stamens showed a marked increase in genes with IM62-like expression, including in background regions where genes are homozygous for SF5 alleles (7% [1,435/19,186] IM62-like in background; yellow and orange points in fig. 6*B*, *D*, and *F* and supplementary table S3, Supplementary Material online).

The fact that misexpression was almost entirely restricted to STE stamens suggests it might be a downstream effect of the hybrid male sterility phenotype. Consistent with this idea, we observed an overrepresentation of stamen-biased genes among the 2,062 genes that were downregulated in STE stamens compared with FER and SF5 stamens (see fig. 4): 83% (1,715) were stamen biased in one or both parents, whereas only 5% (108) were carpel biased in one or both parents (supplementary fig. S4, Supplementary Material online). On the other hand, of the 5,344 genes upregulated in STE stamens compared with FER and SF5 stamens, only 6% (344) were stamen biased in one or both parents and 74% (3,953) were carpel biased (supplementary fig. S4, Supplementary Material online). Moreover, we found that both downregulated STE stamen genes and parental stamen-biased genes were enriched in Gene Ontology (GO) terms related to pollen development (e.g., pollen tube growth, cell tip growth), whereas upregulated STE stamen genes and parental carpel-biased genes were enriched in photosynthesis-related GO terms (supplementary table S4, Supplementary Material online).

### Large Effects of Both *cis-* and *trans-*Regulatory Divergence between Species

**Fig. 7.**
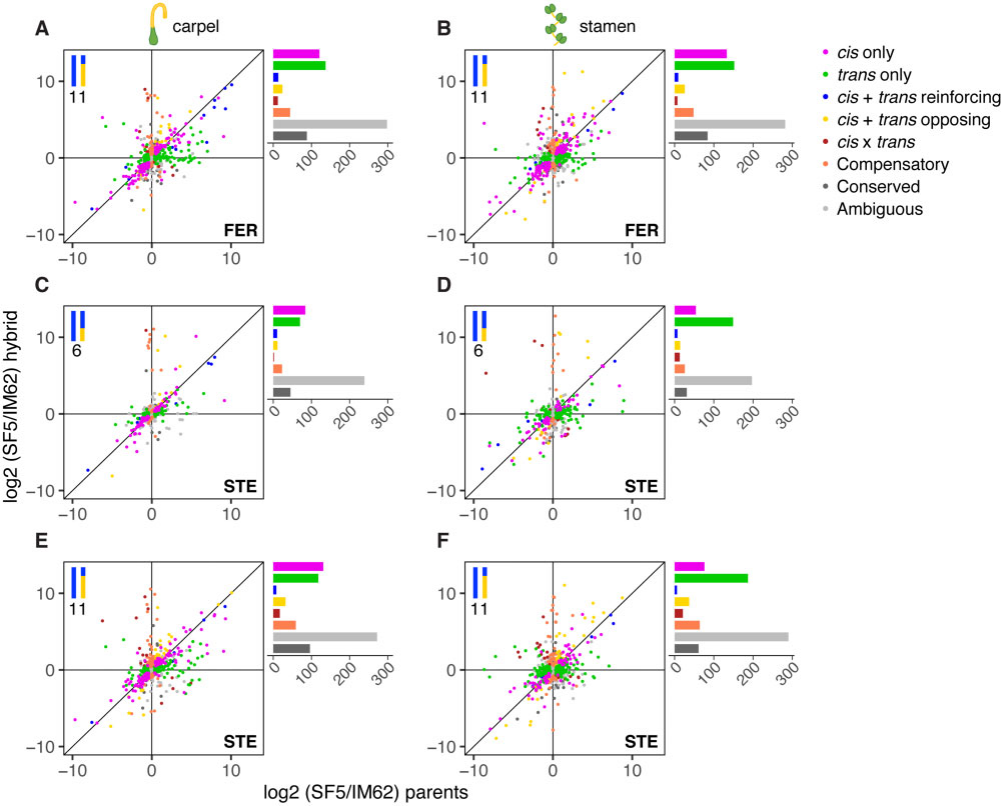
Classification of parental *cis-* and *trans-*regulatory divergence across heterozygous genes in FER and STE introgression hybrids. Points represent relative transcript abundance (log 2 fold-change) between the parents, SF5 and IM62, on the *x*-axis (log 2[SF5/IM62] parents) and relative abundance of SF5- and IM62-specific transcripts in the hybrids on the *y*-axis (log 2[SF5/IM62] hybrid) across (*A*, *B*) FER and (*C*–*F*) STE tissues (i.e., carpels and stamens) for heterozygous genes in the (*C*, *D*) chromosome 6 and (*A*–*D*) chromosome 11 introgression regions. The diagonal line indicates relative parental expression (i.e., log 2[SF5/IM62] parents) and relative allelic expression (i.e., log 2[SF5/IM62] hybrid) are equal. Points that fall along the diagonal line represent genes for whom parental expression differences are entirely explained by *cis*-regulatory divergence. Points are colored by regulatory divergence category. IM62, *Mimulus guttatus* IM62 parent; SF5, *Mimulus nasutus* SF5 parent; FER, fertile RSB_7_ introgression hybrid; STE, sterile RSB_7_ introgression hybrid.

To characterize patterns of regulatory divergence in *Mimulus*, we compared expression differences between species (*M. nasutus* SF5 and *M. guttatus* IM62) with relative expression of SF5 and IM62 alleles (i.e., allele-specific expression) within FER and STE heterozygous introgression genes (fig. 7 and supplementary table S5, Supplementary Material online). Because these heterozygous genes carry an allele from each parent expressed in a common *trans* environment, *cis*-regulatory divergence between SF5 and IM62 is expected to produce biased allele-specific expression in FER and STE tissues. Similarly, divergence in one or more *trans-*factors will affect overall transcript abundance of FER and STE introgression genes without disrupting allele-specific expression. In contrast, compensating *cis* and *trans* mutations may result in expression conservation between SF5 and IM62, despite divergence in the underlying regulatory machinery. To avoid overestimating compensatory changes, in this analysis, we eliminated the log 2 fold-change > 1.25 threshold requirement for detecting expression differences (see Materials and Methods). Consequently, 40–46% (304–566) of the heterozygous introgression genes evaluated in FER and STE tissues exhibited significant (FDR ≤ 0.05) parental expression divergence (supplementary table S5, Supplementary Material online).

Among these divergent heterozygous introgression genes, regulatory divergence in FER and STE tissues was primarily categorized as *trans* only (135–337, 39–60%; green points/bars in fig. 7 and supplementary table S5, Supplementary Material online) or *cis* only (120–215, 23–45%; purple points/bars in fig. 7 and supplementary table S5, Supplementary Material online), with smaller contributions from *cis* + *trans* reinforcing (9–18, 2–4%; blue points/bars in fig. 7 and supplementary table S5, Supplementary Material online), *cis* + *trans* opposing (24–2, 8–9%; yellow points/bars in fig. 7 and supplementary table S5, Supplementary Material online) and *cis* × *trans* (7–34, 2–6%; maroon points/bars in fig. 7 and supplementary table S5, Supplementary Material online). Among the 54–60% (407–731) of heterozygous introgression genes that exhibited parental expression conservation, the majority also exhibited regulatory conservation (278–509, 68–73%; gray points/bars in fig. 7 and supplementary table S5, Supplementary Material online), though 11–13% (45–89) of these genes showed evidence of compensatory *cis–trans* evolution in FER and STE tissues (orange points/bars in fig. 7 and supplementary table S5, Supplementary Material online). Importantly, genes with compensating *cis* and *trans* changes (i.e., *cis* + *trans* opposing, *cis* × *trans*, and compensatory) were no more likely to be misexpressed than genes in other regulatory categories (supplementary fig. S5 and S6 and table S6, Supplementary Material online): in STE stamens, 18% of genes with evidence of compensating *cis* and *trans* divergence were misexpressed compared with 26% of genes with conserved regulation ($\chi_{\left(\text{compensating}\;\mathrm{cis}\;\text{and}\;\mathrm{trans}\;\text{vs}.\;\text{conserved}\right)}^{2}$ = 3.77, *P* value = 0.052) and 18% of genes with other forms of regulatory divergence ($\chi_{\left(\text{compensating}\;\mathrm{cis}\;\text{and}\;\mathrm{trans}\;\text{vs}.\;\text{other}\right)}^{2}$ = 0.01, *P* value = 0.904).

As discussed in the previous section, misexpression is widespread in STE stamens, including in the heterozygous introgressions (fig. 6 and supplementary fig. S5*C*–*F*, Supplementary Material online). If misexpression in STE stamens is due largely to downstream effects of the *hms1–hms2* incompatibility and/or disrupted tissues, using these samples to infer mechanisms of regulatory divergence could be problematic (e.g., if allele-specific expression is affected by altered cellular composition, not by species differences in *cis*- and *trans*-regulation). Although the proportions of genes in each regulatory divergence category are fairly similar across fertile and sterile tissues (fig. 7), we note that inferences from the STE stamen samples (fig. 7*D* and *F*), which show a proportional increase in *trans*-only and decrease in *cis*-only genes, should be made with caution.

### Gene Expression in *hms1* and *hms2* Intervals Points to Candidate Genes

In addition to examining genome-wide patterns of expression, we wanted to investigate individual genes in the *hms1*- and *hms2*-mapped intervals to identify candidates for *Mimulus* hybrid sterility. Our expectation was that the causal genes for severe hybrid male sterility and partial hybrid female sterility would be expressed in stamens, and possibly, in carpels. Additionally, if the *hms1–hms2* incompatibility is mediated by expression changes, we might expect the causal genes to show differences in expression between species and/or between fertile and sterile introgression lines.

Of the 11 genes in the *hms1* interval, we were able to evaluate expression for eight of them (table 1). Among these eight genes, five were expressed at moderate to high levels (fragments per kilobase per million reads sequenced >2) in stamens, with one, Migut.F01606, exhibiting significant (log 2 fold-change > 1.25, FDR ≤ 0.05) stamen bias in SF5, FER, and STE (table 1 and supplementary table S7, Supplementary Material online). Migut.F01606 also showed expression differences between species and between introgression lines: Expression was significantly (log 2 fold-change > 1.25, FDR ≤ 0.05) higher in SF5 and FER stamens compared with IM62 and STE stamens. One of the genes in *hms1*, Migut.F01612, shows copy number variation between species: This gene and its highly similar paralog (Migut.F01618) are present in IM62 but absent in SF5 (Kerwin RE and Sweigart AL, unpublished results). Because of its absence from the genome, expression is precluded in SF5 and FER samples. However, even in IM62 and STE, expression is difficult to gauge because high sequence similarity between Migut.F01612 and Migut.F01618 means that these transcripts did not pass our threshold for unique read mapping. Taken together, these results suggest that Migut.F01606 and Migut.F01612 are the most promising *hms1* candidates.

**Table 1.** Gene Expression at *hms1* and *hms2*.

		Stamen				Carpel			
		IM62	SF5	FER	STE	IM62	SF5	FER	STE
*hms1*	Migut.F01605	—	—	—	—	—	—	—	—
	Migut.F01606	0.2	100.6	50.9	0.8	0.0	0.1	0.1	0.0
	Migut.F01607	17.3	19.2	17.8	17.7	13.2	13.3	12.8	12.1
	Migut.F01608	0.1	0.1	0.3	0.6	4.9	2.1	2.6	3.3
	Migut.F01609	1.0	1.0	0.9	3.4	2.1	3.4	3.3	2.9
	Migut.F01610	—	—	—	—	—	—	—	—
	Migut.F01611	0.3	0.5	0.1	0.8	2.2	1.6	2.3	2.5
	Migut.F01612	—	—	—	—	—	—	—	—
	Migut.F01613	0.8	1.4	1.0	2.5	3.7	4.3	4.1	4.4
	Migut.F01614	0.0	1.9	1.4	1.9	0.0	3.7	3.9	2.4
	Migut.F01615	1.3	1.7	0.9	3.0	4.0	5.4	5.3	4.3
*hms2*	Migut.M00294	3.6	3.1	3.4	6.8	5.2	5.5	5.3	5.5
	Migut.M00295	18.1	16.0	13.5	13.7	11.6	12.7	13.0	11.4
	Migut.M00296	0.4	0.2	0.9	0.7	42.3	38.9	36.6	35.7
	Migut.M00297	11.2	12.2	7.5	0.9	3.3	0.2	0.3	0.3
	Migut.M00298	0.0	0.2	0.1	1.0	2.4	9.5	10.9	10.1

Note.—Table shows absolute transcript abundance in fragments per kilobase per million reads sequenced across the eight genotype-tissue groups in this study for the 16 genes in the mapped regions of *hms1* and *hms2*. Transcript abundances below our threshold of one CPM are denoted by “—.” IM62, *Mimulus guttatus* IM62 parent; SF5, *Mimulus nasutus* SF5 parent; FER, fertile RSB_7_ introgression hybrid; STE, sterile RSB_7_ introgression hybrid. Color indicates expression intensity from low (blue) to high (red).

Of the five genes in the *hms2* interval, three were expressed at moderate to high levels in stamens, with Migut.M00297 showing significant (log 2 fold-change > 1.25, FDR ≤ 0.05) stamen bias in IM62, SF5, and FER samples (table 1 and supplementary table S7, Supplementary Material online). Two genes (Migut.M00296 and Migut.M00298) were significantly (log 2 fold-change > 1.25, FDR ≤ 0.05) carpel biased with very little/no expression in IM62, SF5, FER, and STE stamens, making them unlikely candidates for the *hms2* causal gene. Intriguingly, Migut.M00297 expression was significantly (log 2 fold-change > 1.25, FDR ≤ 0.05) lower in STE stamens compared with IM62, SF5, and FER. This pattern is precisely what is expected if the IM62 *hms1* allele (in the STE chromosome 6 introgression) directly affects expression of the causal *hms2* gene. From this analysis, then, Migut.M00297 emerges as a strong candidate for *hms2*.

## Discussion

Misexpression is a common feature of hybrids between closely related species, but the causal mechanisms and evolutionary significance are not always clear. Aberrant patterns of gene expression in sterile or inviable hybrids could be due to regulatory incompatibilities—which would implicate divergence in regulatory networks as a driver of reproductive isolation—or to the downstream effects of disrupted tissues. In this study, we took advantage of introgression hybrids between closely related *Mimulus* species to distinguish between the potential causes of misexpression. Although we discovered substantial regulatory divergence between *M. guttatus* and *M. nasutus*, the fact that a heterozygous introgression spanning most of chromosome 11 caused almost no misexpression suggests that regulatory incompatibilities are not pervasive. Instead, we found that misexpression was confined to the affected tissues (i.e., stamens) of individuals carrying a sterility-causing introgression on chromosome 6, suggesting widespread effects of the *hms1–hms2* incompatibility on gene regulation.

Despite the recentness of their split (∼200 ka, Brandvain et al. 2014), *M. guttatus* and *M. nasutus* showed considerable variation in gene expression, with ∼10% of genes differentially expressed between species in stamens and/or carpels. Extensive expression divergence over short evolutionary timescales has also been observed in animals (Rottscheidt and Harr 2007; Renaut et al. 2009; Coolon et al. 2014) and other plant systems (Fujimoto et al. 2011; Combes et al. 2015). However, we note that because nucleotide divergence between these *Mimulus* species barely exceeds diversity within *M. guttatus* (*d_s_* = 4.94% and *π_s-M. guttatus_* = 4.91%, Brandvain et al. 2014), much of the observed expression variation between *M. guttatus* IM62 and *M. nasutus* SF5 might be segregating within species. We also observed more tissue-biased gene expression in *M. guttatus* than in *M. nasutus* (see fig. 3: IM62 has ∼10% more genes with carpel- and stamen-biased expression). An intriguing possibility is that this difference might reflect divergence in reproductive traits associated with the species’ distinct mating systems (selfing vs. outcrossing).

Our targeted look at gene expression in two heterozygous introgressions suggests that regulatory differences between *Mimulus* species are due to divergence in both *trans*-factors and *cis*-regulatory sequences. This finding runs counter to the expectation that interspecific regulatory variation should be influenced primarily by changes to *cis*-regulatory sequences, which have fewer pleiotropic effects (Wray et al. 2003; Wittkopp et al. 2008). However, it is in agreement with several recent studies showing that *cis*-changes do not always predominate between closely related species (McManus et al. 2010; Meiklejohn et al. 2014; Combes et al. 2015; Guerrero et al. 2016; Metzger et al. 2017). When two lineages have split only recently, some of their regulatory differences might still be polymorphic within species, with purifying selection having had insufficient time to remove deleterious *trans*-regulatory variants. This argument, along with evidence that mutations in *trans*-factors arise more frequently (Landry et al. 2007), might explain the higher than expected contribution of *trans*-acting factors to regulatory variation observed within species (Wittkopp et al. 2008; Emerson et al. 2010), as well as between closely related species. In the case of the two *Mimulus* species studied here, an additional factor may affect *trans*-regulatory divergence: Deleterious mutations might be particularly likely to accumulate in *M. nasutus* because of its shift to self-fertilization. Indeed, this species shows genomic signatures that indicate a reduction in the efficacy of purifying selection (Brandvain et al. 2014), an expected outcome of the lower effective population size and recombination rate that accompanies the evolution of selfing (Nordborg 2000; Charlesworth and Wright 2001).

In addition to *cis*- and *trans*-only regulatory divergence between these *Mimulus* species, our analyses of the two heterozygous introgressions uncovered some evidence of compensatory *cis–trans* evolution. Interestingly, however, we found little indication that this process acts as a general driver of regulatory incompatibilities. Genes with evidence of compensating *cis* and *trans* (i.e., *cis* + *trans* opposing, *cis* × *trans*, and compensatory) divergence were no more likely to be misexpressed in STE stamens than genes with conserved regulation (supplementary fig. S6 and table S6, Supplementary Material online). This result is somewhat surprising because compensatory changes within species are expected to cause mismatches between heterospecific *cis*- and *trans*-regulators in hybrids, leading to aberrant gene expression and, potentially, hybrid dysfunction (Landry et al. 2005). In support of this idea, several studies have shown that genes with *cis*- and *trans*-variants that act in opposing directions (i.e., *cis* + *trans* opposing, *cis* × *trans*, and compensatory) are enriched among genes that are misexpressed in hybrids (Tirosh et al. 2009; McManus et al. 2010; Schaefke et al. 2013; Mack et al. 2016). On the other hand, it has been argued that *cis* × *trans* effects might often be inflated (Fraser 2019), and, in some cases, the association is missing altogether (Bell et al. 2013; Coolon et al. 2014).

What is clear from our study is that species divergence in *cis*- and *trans*-regulatory elements cannot explain the vast majority of the gene misexpression we observe in *Mimulus* introgression hybrids. Instead, the fact that widespread misexpression is confined to sterile tissues suggests it is a downstream consequence of the *hms1–hms2* incompatibility. Indeed, introgressing a 7-Mb genomic segment with the *hms1* incompatibility allele from *M. guttatus* into *M. nasutus* has profound effects on male fertility, with STE individuals producing few pollen grains, nearly all of which are inviable. Coincident with this male sterility, STE stamens showed dramatic expression differences when compared with parental lines, with 22% of all genes misexpressed (*N* = 20,431; fig. 6 and supplementary table S3, Supplementary Material online). In stark contrast, introgressing a genomic segment from chromosome 11 that is much larger in size (23 Mb) but does not carry any known hybrid incompatibility alleles, resulted in only a single misexpressed transcript in stamens (i.e., in FER individuals; fig. 5 and supplementary table S3, Supplementary Material online). The fact that neither introgression showed strong effects on expression in carpels suggests that partial hybrid female sterility either has little impact on gene regulation or manifests later in development.

Our study highlights the challenge of establishing a causal link between divergent regulatory alleles and the evolution of hybrid incompatibilities. When hybrid misexpression is discovered, it is often confounded with the phenotypic effects of hybrid dysfunction, such as defective tissues and/or disrupted development (Ortíz-Barrientos et al. 2007; Wei et al. 2014). For example, when normally inviable F_1_ hybrid males between *Drosophila melanogaster* and *Drosophila simulans* are rescued by a mutation in the hybrid incompatibility gene *Hmr*, gene expression in larvae becomes much more similar to parents (Wei et al. 2014). Like with the *hms1–hms2* incompatibility in *Mimulus*, this result suggests a large effect of the *Hmr* gene on genome-wide hybrid misexpression in *Drosophila*. A similar result was also seen in a previous study of *M. nasutus–M. guttatus* F_2_ hybrids: The number of differentially expressed genes between parents and lethal F_2_ seedlings that lack chlorophyll due to a two-locus hybrid incompatibility is much higher than between parents and viable F_2_ seedlings (Zuellig and Sweigart 2018). Taken together, these studies suggest that caution should be taken when assigning a cause to hybrid gene misexpression. At the same time, it is important to note that our results do not rule out regulatory divergence as a cause of *Mimulus* hybrid incompatibilities in particular cases, including *hms1* and *hms2*.

An additional outcome of our analyses is a shortened list of likely candidate genes for causing the *hms1–hms2* incompatibility. Previously, we mapped *hms1* to an interval containing 11 annotated genes with three strong functional candidates: *Migut.F01605*, *Migut.F01606*, and *Migut.F01612* (Sweigart and Flagel 2015). The first two are tandem duplicates of *SKP1*-like genes, which form part of the SKP1–Cullin–F-box protein E3 ubiquitin ligase complex that regulates many developmental processes including the cell cycle (Hellmann and Estelle 2002). Although we did not detect *Migut.F01605* expression in any sample (potentially calling into question its functionality), *Migut.F01606* remains a strong candidate. Expression of this gene was significantly (log 2 fold-change > 1.25, FDR ≤ 0.05) stamen biased in FER, STE, and SF5 and significantly higher in SF5 and FER than IM62 or STE (table 1). If *Migut.F01606* is causal for *hms1*, the fact that the normally expressed SF5 allele is significantly reduced in heterozygous STE individuals suggests that the IM62 allele interferes with its expression. An alternative possibility is that reduced expression of *Migut.F01606* in STE is simply a downstream effect of the hybrid male sterility phenotype. *Migut.F01612*, an F-box gene, also remains a strong candidate for *hms1*. Although the RNAseq results provided little additional insight into the function of this gene (transcript abundance did not pass our expression threshold of one CPM in any genotype-tissue group), we have observed its expression in IM62 via reverse transcription polymerase chain reaction (data not shown) and its absence from the SF5 genome is notable.

At *hms2*, expression patterns of *Migut.M00297* strengthen it as a candidate. This gene encodes the second-largest subunit (*RPB2*) of RNA Polymerase II—the multi-subunit enzyme responsible for mRNA transcription (Woychik and Hampsey 2002; Hahn 2004). In most flowering plant species, *RPB2* is a single copy gene. However, within the asterid clade, two distinct paralogs (*RPB2*-*i* and *RPB2*-*d*) are present, having been retained following an ancient duplication event (Oxelman et al. 2004; Luo et al. 2007). In all asterid species that have been investigated, the expression pattern of *RPB2-i* suggests that it is restricted to male reproductive structures (e.g., stamen and pollen) (Oxelman et al. 2004; Luo et al. 2007). In our experiment, expression of *Migut.M00297*, which encodes the *RPB2-i* paralog, was significantly (log 2 fold-change > 1.25, FDR ≤ 0.05) stamen biased in both parents and in FER but significantly underexpressed in STE individuals (table 1). Although this is the pattern expected if the IM62 *hms1* allele (in the STE chromosome 6 introgression) directly affects the expression of the causal *hms2* gene, it might also arise as a byproduct of *hms1–hms2* sterility. Of course, an important consideration is that, for both *hms1* and *hms2*, the difference between compatible and incompatible alleles might have nothing to do with transcription. For each of these loci, then, additional approaches such as transformation experiments will be needed to identify the causal genes.

In addition to the main findings just discussed, our study has revealed a dramatic suppression of recombination in the RSB introgression population. Despite eight rounds of backcrossing to *M. nasutus* SF5, the heterozygous introgressions on chromosomes 6 and 11 remain quite large (7 and 23 Mb, respectively). With uniform recombination rates and Mendelian transmission, FER and STE individuals are expected to be heterozygous along ∼0.2% of their genome, which equates to a maximum introgression size of ∼0.625 Mb (*M. guttatus* genome ∼312 Mb). Suppressed recombination rates on chromosome 6 were previously observed in an earlier generation of the RSB population (Sweigart et al. 2006). At the time, we speculated that low recombination might be a direct cause of the *hms1–hms2* incompatibility—perhaps due to a meiotic defect. However, follow-up work performing testcrosses with F_2_ hybrids that carried either incompatible or compatible genotypes at *hms1* and *hms2* showed no effect of the *hms1–hms2* incompatibility on recombination rates (data not shown). Additionally, we have observed a similar reduction in recombination in heterospecific introgressions when attempting to generate nearly isogenic lines using other *Mimulus* accessions that lack the *hms1–hms2* incompatibility. A possible explanation for the suppressed recombination on chromosomes 6 and 11 is that local sequence diversity affects recombination in *Mimulus*. Nucleotide diversity between chromosome homologs is much higher in heterospecific introgressions than in adjacent isogenic regions. Thus, if sequence diversity affects the likelihood of DNA double-strand breaks and/or crossover events, as it does in mice (Li et al. 2019), we would expect much lower recombination in the heterozygous introgressions. Given the extraordinarily high nucleotide diversity within *M. guttatus* (Brandvain et al. 2014; Puzey et al. 2017), if this explanation is correct, we might expect extensive natural variation in recombination rates even within species.

## Materials and Methods

### Plant Lines and Growth Conditions

This study focuses on *M. guttatus* and *M. nasutus*, two closely related species that diverged roughly 200,000 years ago (Brandvain et al. 2014). Previous work identified two nuclear incompatibility loci, *hms1* and *hms2*, that cause nearly complete (>90%) male sterility and partial female sterility in a fraction of F_2_ hybrids between an inbred line of *M. guttatus* from Iron Mountain, Oregon (IM62), and a naturally inbred *M. nasutus* line from Sherar’s Falls, Oregon (SF5) (Sweigart et al. 2006). We generated an introgression population carrying incompatible (IM62) and compatible (SF5) *hms1* alleles in a common genetic background fixed for the incompatible (SF5) *hms2* allele, through multiple rounds of selection for pollen sterility and backcrossing to the recurrent SF5 parent (Sweigart et al. 2006). Briefly, *M. nasutus* SF5 and *M. guttatus* IM62 were intercrossed (with SF5 as the maternal parent) to create an F_1_ hybrid that was backcrossed to SF5 (with the F_1_ as the maternal parent), producing a first-generation backcross (BC_1_) population that segregates four *hms1–hms2* genotypes (fig. 1). Next, a pollen sterile individual selected from the BC_1_ population was backcrossed to SF5, yielding the first generation of an introgression population dubbed *r*ecurrent *s*election with *b*ackcrossing (RSB_1_) (Sweigart et al. 2006). This selective backcrossing scheme was repeated six more times, producing an RSB_7_ population that segregates ∼1:1 for two genotypes: pollen STE individuals carrying the incompatible IM62 allele at *hms1*and pollen FER individuals homozygous for the compatible SF5 allele at *hms1*, both in a genetic background fixed for the incompatible (SF5) *hms2* allele (fig. 1).

All plants were grown in a growth chamber at the University of Georgia. Seeds were sown into 2.5-in. pots containing Fafard 3B potting mix (Sun Gro Horticulture, Agawam, MA), stratified for 7 days at 4 °C, then transferred to a growth chamber set to 22 °C day/16 °C night, 16-h day length. Plants were bottom-watered daily and fertilized as needed using Jack’s Professional Blossom Booster (J.R. Peter’s, Inc., Allentown, PA). To identify FER and STE RSB_7_ genotypes prior to bud formation, plants were genotyped at markers flanking *hms1* and *hms2*. To verify the fertility phenotypes, the first flower on each RSB_7_ plant was allowed to self-pollinate. Within 3–5 days postanthesis, fertilized fruits (on FER plants) begin to mature and plump, whereas the unfertilized fruits (on STE plants) remain immature and small, making it easy to differentiate the two phenotypic classes.

### Sample Collection and Transcriptome Sequencing

For this study, we generated 24 whole transcriptome libraries: three biological replicates each of two tissue types (stamens and carpels) from four genotypes that vary at *hms1* and *hms2* (*M. guttatus* IM62, *M. nasutus* SF5, RSB_7_ FER, and RSB_7_ STE; fig. 1 and supplementary table S1, Supplementary Material online). To identify FER and STE individuals from the RSB_7_ population prior to flowering, we genotyped the plants using PCR-based markers flanking *hms1* and *hms2*. To collect sufficient tissue for each biological replicate, we carefully dissected 8–24 preanthesis floral buds and transferred carpel and stamen tissue into separate 1.5-ml microcentrifuge tubes partially submerged in liquid nitrogen (supplementary table S1, Supplementary Material online). We extracted RNA using a QuickRNA Miniprep Kit (Zymo Research, Irvine, CA) then measured RNA concentration using a Qubit RNA BR (Broad-Range) Assay Kit and a Qubit 2.0 Fluorometer (Thermo Fisher Scientific Inc., Waltham, MA). We shipped RNA samples overnight on dry ice to the Duke Center for Genomic and Computational Biology (Durham, NC), where the Sequencing and Genomic Technologies core checked RNA quality using an Bioanalyzer 2100 (Agilent Technologies, Santa Clara, CA), constructed sequencing libraries using a KAPA Stranded mRNA-Seq Kit (F. Hoffmann-L Roche, Basel, Switzerland), and sequenced all 24 libraries on a single lane of HiSeq 4000 (Illumina, Inc. San Diego, CA), producing 50-base pair single-end reads (supplementary table S1, Supplementary Material online). The raw fastq files are available to download from the Sequence Read Archive database (supplementary table S8, Supplementary Material online).

### Pseudoreference Genome Construction and Competitive Transcriptome Alignment

An important consideration for genomic and transcriptomic analyses is potential mapping bias introduced when aligning reads from one species against a reference from another species (Degner et al. 2009). The four genotypes in this study represent two pure species, *M. guttatus* IM62 and *M. nasutus* SF5, as well as FER and STE individuals from the RSB_7_ population that are expected to carry SF5 alleles across >90% of their genomes. Previous work has shown that nucleotide divergence between *M. guttatus* and *M. nasutus* is substantial (*d_s_* = 4.94%, see Brandvain et al. 2014). Therefore, aligning SF5, FER, and STE reads against the *M. guttatus* v2.0 reference (which is based on the IM62 accession; Hellsten et al. 2013) is likely to introduce mapping bias: Reads from nonreference alleles may align incorrectly, nonuniquely, or not at all due to mismatch errors. To ameliorate this issue, we constructed a diploid *M. guttatus* IM62–*M. nasutus* SF5 pseudoreference genome and competitively mapped all RNAseq reads against it.

First, we constructed a *M. nasutus* pseudoreference genome using publicly available SF5 whole genome (gDNA) sequence data. Using the fastq-dump command from the NCBI toolkit, we retrieved the SF5 gDNA fastq files from the NCBI Sequence Read Archive database (SRR400478). To prepare the 75-bp paired-end sequences for alignment, we trimmed adapters and low-quality bases, then filtered out processed reads shorter than 50 bp using Trimmomatic (Bolger et al. 2014). We aligned the trimmed 50- to 75-bp paired-end reads to the *M. guttatus* v2.0 reference genome using BWA-MEM (Li and Durbin 2009; Li 2013). We removed optical and PCR duplicates from the initial SF5 alignment using Picard MarkDuplicates (http://broadinstitute.github.io/picard; last accessed December 20, 2019) then filtered out reads with an alignment quality below Q20 with SAMtools view command (Li et al. 2009). Next, we generated a set of high-quality SNPs for *M. nasutus* SF5 pseudoreference genome construction. Using GATK HaplotypeCaller in GVCF mode followed by GATK GenotypeGVCFs, we identified phased SNP and insertion/deletion (indel) variants from the SF5 alignment (McKenna et al. 2010; Poplin et al. 2017). From there, we extracted biallelic SNPs with GATK SelectVariants and filtered out sites with a mapping quality below 40 or quality by depth below two using GATK VariantFiltration. Using the filtered SNPs, we created a *M. nasutus* SF5 pseudoreference genome with GATK FastaAlternateReferenceMaker. Finally, we constructed a diploid *M. guttatus* IM62–*M. nasutus* SF5 pseudoreference genome by appending allelic (i.e., IM62 or SF5) identifiers onto the chromosome names in the *M. guttatus* v2.0 reference and *M. nasutus* SF5 pseudoreference fasta files, then merging them manually into a single file. We similarly created a diploid annotation file by appending allelic identifiers onto chromosome, gene, and transcript names, then combining the two files.

To generate RNAseq alignments, we mapped all four genotypes (IM62, SF5, FER, and STE) to the diploid pseudoreference genome. Prior to mapping, we trimmed adapter sequences and low-quality bases from the raw RNAseq reads, then filtered out reads shorter than 36 bp using Trimmomatic (Bolger et al. 2014) (supplementary table S1, Supplementary Material online). We mapped the resulting 36–50-bp single-end RNAseq reads using STAR (Dobin et al. 2013; Dobin and Gingeras 2015). Reads that overlap a SNP are expected to map uniquely to an SF5 or IM62 allele in the pseudoreference genome, whereas reads that do not overlap a SNP will map equally well to both alleles. We limited each read to a maximum of two alignments by specifying –outFilterMultimapNmax 2. With this approach, >98.6% of reads mapped to a single genomic location, whether they mapped uniquely to one allele or not. For reads with two alignments, we randomly designated one as primary by specifying –outMultimapperOrder Random. Then, we removed secondary alignments and unmapped reads using SAMtools view, leaving just unique (i.e., allele specific) and primary alignments. We filtered out optical and PCR duplicates with Picard MarkDuplicates, then parsed intron-spanning reads into exon segments and trimmed off bases that extended into intronic regions using GATK SplitNCigarReads (McKenna et al. 2010). Finally, with SAMtools view, we filtered based on mapping quality to obtain high-quality alignments with two kinds of reads: 1) allele-specific and primary mapping (i.e., total), using a Q20 threshold or 2) just allele specific, using a Q60 threshold, which designates unique alignments.

### Genotyping and Determination of Heterozygous Introgression Boundaries

To determine the heterozygous introgression boundaries for the FER and STE samples, we counted allele-specific reads from the Q60 alignments using HTSeq-count (Anders et al. 2015), with the nonunique parameter set to none, and converted the read counts to binary presence/absence values for genotyping. First, to ensure that only expressed genes were used for genotyping, we set read counts below 10 to 0. Then, we converted all read counts above 0 to 1. Using the binary read count scores from the IM62 and SF5 alleles, we assigned one of four genotypes to each gene in each sample: 1) IM62 (IM62 = 1, SF5 = 0), 2) HET (IM62 = 1, SF5 = 1), 3) SF5 (IM62 = 0, SF5 = 1), and 4) nonexpressed (IM62 = 0, SF5 = 0). To obtain a single genotype call per gene for FER and STE individuals, we pooled the six biological replicates from each line. The majority of FER and STE genotype calls were HET or SF5, though a few were IM62. The erroneous IM62 genotype assignments could be caused by a number of reasons, including 1) the SF5 allele was not expressed our samples, 2) sequencing error caused an SF5-derived read to match IM62, or 3) the gene is absent from the SF5 genome. We dropped the erroneous IM62 genotype calls, which eliminated 26 and 39 FER and STE genes, respectively, resulting in genotype assignments for 18,680 and 18,675 genes across the 14 *Mimulus* chromosomes.

### Differential Gene Expression Analysis and Gene Expression Category Assignment

To quantify total transcript abundance, we counted total (i.e., allele-specific and primary mapping) reads from the processed Q20 alignments using HTSeq-count (Anders et al. 2015), with the nonunique parameter set to all. We used these raw read counts to perform differential gene expression analysis in edgeR (Robinson et al. 2010). To restrict comparisons to genes expressed in at least one genotype-tissue group, we only analyzed genes with at least one read CPM in three or more of the 24 libraries. This filtering step removed 7,188 genes, resulting in a set of 20,952 expressed genes, 20,431 of which are distributed across the 14 *Mimulus* chromosomes. We normalize libraries based on RNA composition with the calcNormFactors function, using the default trimmed mean of M-values method. To compare gene expression across the 24 samples in our data set, we used the plotMDS function in edgeR to generate a MDS plot, which is a type of unsupervised clustering plot (fig. 2). The distance between two points in an MDS plot represents the leading log-fold-change (i.e., largest absolute log-fold-change) between that sample pair. To test for differences in gene expression across the genotype-tissue groups, we conducted generalized linear model (GLM) analyses using a quasi-likelihood (QL) approach in edgeR. This method is flexible and permits any combination of sample comparisons to be made. First, we generated an experimental design matrix describing the eight genotype-tissue groups using the model.matrix function, then fitted it to a QL GLM framework using the glmQLFit function in edgeR. To identify genes with a log 2 fold-change > 1.25 between comparisons, we used the glmTreat function in edgeR, which performs threshold hypothesis testing on the GLM specified by the glmQLFit function. This is a rigorous statistical test that detects expression differences greater than the specified threshold value by evaluating both variance and magnitude of change in expression, then applies FDR *P* value corrections. We categorized genes as significantly differentially expressed between two groups if the log 2 FC > 1.25 and the FDR-corrected *P* value ≤ 0.05.

We categorized gene expression in the STE and FER RSB_7_ individuals based on interspecific expression differences between IM62 and SF5, as well as differences between the RSB_7_ individual and its two parents (supplementary table S2, Supplementary Material online). This resulted in eight expression categories: 1) Similar: the parents and RSB_7_ all have similar expression; 2) SF5-like (similar): RSB_7_ expression is similar to SF5 and significantly different than IM62. Expression does not differ between parents; 3) SF5-like (divergent): RSB_7_ expression is similar to SF5 and significantly different from IM62. Expression differs significantly between parents; 4) IM62-like (similar): RSB_7_ expression is similar to IM62 and significantly different from SF5. Expression does not differ between parents; 5) IM62-like (divergent): RSB_7_ expression is similar to IM62 and significantly different from SF5. Parents differ significantly; 6) Intermediate (divergent): RSB_7_ expression falls within the parental range. Expression differs significantly between parents; 7) Misexpressed (similar): RSB_7_ expression is higher or lower than both parents. Expression does not differ between parents; 8) Misexpressed (divergent): RSB_7_ expression is higher or lower than both parents. Expression differs significantly between parents.

### Allele-Specific Expression Analysis and Regulatory Divergence Category Assignment

By comparing gene expression differences between species to allele-specific expression within interspecific hybrids, regulatory divergence can partitioned among contributing *cis* and *trans* components (Wittkopp et al. 2004). We utilized the allele-specific read counts to perform allele-specific expression analysis in edgeR (Robinson et al. 2010). To measure allele-specific expression bias in heterozygous genes within the introgression regions across STE and FER stamens and carpels, we compared proportions of reads that mapped to IM62 versus SF5 alleles. To limit our analyses to expressed genes, we eliminated genes with fewer than one allelic CPM in each genotype-tissue group. To test for bias in allele-specific expression across FER and STE tissues, we fitted our allele-specific count data to a GLM with the glmQLFit function, then performed QL ratio F-tests with the glmQLFTest function in edgeR. We categorized genes as having significant bias in allele-specific expression if the FDR-corrected *P* value ≤ 0.05.

We quantified total (*cis* and *trans*) interspecific regulatory divergence across stamens and carpels as relative transcript abundance (log 2 fold-change) between SF5 and IM62 (pFC). Significant (FDR ≤ 0.05) differences in parental gene expression (i.e., significant pFC) were considered evidence of total (*cis* and *trans*) regulatory divergence. For the purpose of regulatory divergence categorization, pFC tests were performed using glmQLFTest function in edgeR, eliminating the log 2 fold-change > 1.25 threshold used for differential gene expression analysis. For heterozygous genes in the chromosome 6 and chromosome 11 introgressions, we were able to estimate *cis-*regulatory divergence as the relative abundance of allele-specific transcripts (log 2 fold-change) in the carpels and stamens of FER and STE introgression hybrids (aFC). A significant (FDR ≤ 0.05) imbalance in the ratio of SF5 versus IM62 alleles in the introgression hybrids (i.e., significant aFC) was considered evidence of *cis-*regulatory divergence. Genes with significant pFC or significant aFC were analyzed for significant *trans* effects by comparing pFC and aFC using Student’s *t*-test followed by FDR *P* value correction, implemented using the t.test and p.adjust functions in R (v3.6.1). Significant (FDR ≤ 0.05) differences between pFC and aFC were considered evidence for *trans* divergence. Genes with evidence of both *cis-* and *trans-*regulatory divergence were further categorized according to whether *cis* and *trans* effects acted in the same or opposite directions.

We partitioned heterozygous FER and STE carpel and stamen genes into different regulatory divergence categories using the following rules (modeled after McManus et al. 2010; Mack et al. 2016): 1) *cis* only: pFC and aFC significant, *trans* not significant; 2) *trans* only: pFC significant, aFC not significant; 3) *cis + trans:* pFC, aFC, and *trans* significant; pFC and aFC have same sign (i.e., the species with higher expression contributes higher expressing *cis-*allele in hybrids); *cis + trans* was further divided into *cis + trans* reinforcing (magnitude of pFC is greater than aFC) and *cis + trans* opposing (magnitude of pFC is less than aFC); 4) *cis* × *trans*: pFC, aFC, and *trans* significant; pFC and aFC have opposite signs (i.e., the species with higher expression contributes lower expressing *cis-*allele in hybrids); 5) compensatory: pFC not significant, aFC and *trans* significant; 6) conserved: pFC and aFC not significant; and 7) ambiguous: any other combination of pFC, aFC, and *trans* (which have no clear interpretation).

### GO Enrichment Analysis

We performed GO term enrichment analysis using the PlantRegMap online server (http://plantregmap.cbi.pku.edu.cn/index.php; last accessed January 19, 2020). To identify overrepresented GO terms within sets of differentially expressed genes, *P* value ≤ 0.01 was chosen as the significance threshold. We used the 20,431 genes expressed across the 14 *Mimulus* chromosomes as the background gene list for GO term enrichment analysis.

## Supplementary Material

Supplementary data are available at *Molecular Biology and Evolution* online.

## Supplementary Material

msaa071_Supplementary_Data
