# Supplementary material for: Rampant Misexpression in a Mimulus (Monkeyflower) Introgression Line Caused by Hybrid Sterility, Not Regulatory Divergence
Source: Mol Biol Evol. 2020 Mar 20;37(7):2084–98. doi: 10.1093/molbev/msaa071 (PMC7306685; doi:10.1093/molbev/msaa071)
Supplement: msaa071_Supplementary_Data [file msaa071_supplementary_data.zip › msaa071-Suppl_Data/MBEresub_Supp_fig&table_legends.pdf]

SUPPLEMENTAL FIGURE AND TABLE LEGENDS

Figure S1. Genome-wide parental ancestry in FER and STE introgression hybrids. Plots show the proportion of heterozygous SF5-IM62 (H, green) and homozygous SF5 (N, blue) ancestry in 50 Kb bins across the 14 chromosomes for (A) FER and (B) sterile genotypes. The length of each chromosome represents physical size based on the *M. guttatus* v2.0 assembly (Hellsten *et al.*, 2013). Genotypes were assigned using allele-specific reads from FER and STE (see Methods for details). Chromosomal regions without reliable allele-specific reads are represented by white space. FER and STE genotypes carry a non-sterility-causing heterozygous introgression along 23 Mb of chromosome 11 associated with a meiotic drive element (*D*). Additionally, STE genotypes carry a 7 Mb sterility-causing heterozygous introgression around *hms1* on chromosome 6. IM62 = *M. guttatus* IM62 parent, SF5 = *M. nasutus* SF5 parent, FER = fertile RSB_7 introgression hybrid, STE = sterile RSB_7 introgression hybrid

Figure S2. Interspecific regulatory divergence between SF5 and IM62. Points represent relative transcript abundance (log2 fold-change) between the parents, SF5 and IM62, on the y-axis (log2[SF5/IM62]), and average transcript abundance on the y-axis (log2 counts-per-million [CPM]) in (A) carpels and (B) stamens. Points are colored based on whether transcript abundance is similar (grey), significantly (log2 fold-change >1.25, FDR ≤ 0.05) downregulated (blue) or upregulated (red) in SF5 versus IM62. Dashed lines at 1.25 and -1.25 demarcate the relative transcript abundance thresholds for upregulation and downregulation, respectively

Figure S3. Genome-wide distribution of differentially expressed genes in STE stamens. Relative transcript abundance (log2 fold-change) across the 14 *Mimulus* chromosomes for the 7406 genes significantly (log2 FC > 1.25, FDR ≤ 0.05) differentially expressed in STE stamens compared to FER and SF5 stamens. To quantify the magnitude of expression difference in the heterozygous introgressions and homozygous background regions, we compared transcript abundance in STE stamen to the SF5-IM62 mid-parent value or SF5 parent, respectively. Grey histograms represent relative density of expressed genes measured in 50Kb bins across the 14 chromosomes. Green bars demarcate the heterozygous introgressions on chromosome 6 and chromosome 11. IM62 = *M. guttatus* IM62 parent, SF5 = *M. nasutus* SF5 parent, FER = fertile RSB_7 introgression hybrid, STE = sterile RSB_7 introgression hybrid

Figure S4. Enrichment in tissue-bias among differentially expressed genes in STE stamen. Points show pattern of parental tissue-biased expression (log2 fold-change) for genes that were significantly (log2 FC > 1.25, FDR ≤ 0.05) upregulated (red), downregulated (blue) or similar (grey) in STE stamens compared to FER and SF5 stamens. Among the 2062 genes downregulated in STE stamens, 83% (1715) and 5% (108) were stamen- and carpel-biased in the parents (i.e. SF5 and IM62). Among the 5344 genes upregulated in STE stamens, 6% (344) and 74% (3953) were stamen- and carpel-biased in the parents. IM62 = *M. guttatus* IM62 parent, SF5 = *M. nasutus* SF5 parent, STE = sterile RSB_7 introgression hybrid

Figure S5. Pattern of gene expression across regulatory divergence categories in heterozygous introgression genes in FER and STE hybrids. Points represent relative transcript abundance (log2 fold-change) between the parents, SF5 and IM62, on the x-axis (log2[SF5/IM62] parents) and relative abundance of SF5- and IM62-specific transcripts in the hybrids on the y-axis (log2[SF5/IM62] hybrid) across (A-B) FER and (C-F) STE tissues (i.e. carpels and stamens) for heterozygous genes in the (C-D) chromosome 6 and (A-B, C-D) chromosome 11 introgression regions. The diagonal line denotes equal relative parental expression (i.e. log2[SF5/IM62] parents) and relative allelic expression (i.e. log2[SF5/IM62] hybrid). Points that fall along the diagonal line represent genes for whom parental expression differences are entirely explained by *cis*-regulatory divergence. This figure is identical to Figure 7 except here points are colored by expression category rather than regulatory divergence category (see Table S2 for description). IM62 = *M. guttatus* IM62 parent, SF5 = *M. nasutus* SF5 parent, FER = fertile RSB_7 introgression hybrid, STE = sterile RSB_7 introgression hybrid

Figure S6. Proportion of heterozygous introgression genes in each regulatory divergence category exhibiting different expression patterns in FER and STE tissues. Plots shows gene expression pattern across different regulatory divergence categories for heterozygous introgression genes in FER (top) and STE (bottom) carpels (left) and stamens (right). FER = fertile RSB_7 introgression hybrid, STE = sterile RSB_7 introgression hybrid

Table S1. Sampling, sequencing and alignment summary for the 24 samples in this study. IM62 = *M. guttatus* IM62 parent, SF5 = *M. nasutus* SF5 parent, FER = fertile RSB_7 individual, STE = sterile RSB_7 individual, % allele-specific = percentage of reads that map uniquely to an SF5 or IM62 allele in the diploid pseudoreference genome alignment, % non-allele-specific = percentage of reads that map to single genomic location in the diploid pseudoreference alignment, but map equally well to both alleles, % SF5 background/% SF5 chr 6/% SF5 chr11 = percentage of allele-specific aligning to the SF5 allele in the background region, chromosome 6 introgression, or chromosome 11 introgression

Table S2. Gene expression category assignment. Gene expression in FER and STE tissues was categorized based on three pairwise comparisons: (i) SF5 vs IM62, (ii) RSB vs SF5, and (iii) RSB vs IM62. S/NS = transcript abundance is significantly different (log2 fold-change > 1.25, FDR ≤ 0.05, S) or not (NS) between samples for a comparison, UP/DOWN = relative transcript abundance (log2 fold-change) is >0 (UP) or <0 (DOWN) for a comparison (i.e. expression is higher (UP) or lower (DOWN) in RSB compared to SF5/IM62), IM62 = *M. guttatus* IM62 parent, SF5 = *M. nasutus* SF5 parent, FER = fertile RSB_7 introgression hybrid, STE = sterile RSB_7 introgression hybrid, RSB = FER or STE RSB_7 introgression hybrid

Table S3. Expression category gene counts. Table reports counts and percentages of genes in the heterozygous introgressions and background regions that fall into one of eight different expression categories across FER and STE carpels and stamens (see Figures 5 and 6). Also listed are the counts of genes exhibiting parental expression divergence (SF5-IM62 divergent), genes parental expression conservation (SF5-IM62 similar), and total expressed genes (Total) in each genomic region across the different genotype-tissue groups. Percentages of Misexpressed (divergent), SF5-like (divergent), Intermediate, and IM62-like (divergent) are calculated based on the number of genes exhibiting parental expression divergence (SF5-IM62 divergent), whereas percentages of Misexpressed (similar), SF5-like (similar), IM62-like (similar) and Similar are based on genes exhibiting parental expression conservation (SF5-IM62 similar). IM62 = *M. guttatus* IM62 parent, SF5 = *M. nasutus* SF5 parent, FER = fertile RSB_7 introgression hybrid, STE = sterile RSB_7 introgression hybrid

Table S4. GO term enrichment. Shown are the top 10 biological process-related GO terms for four categories of genes: (i) downregulated in STE stamens compared to FER and SF5 (ii) stamen-biased in SF5 and IM62 (i.e. Parental stamen-biased), (iii) upregulated in STE stamens compared to FER and SF5, and (iv) carpel-biased in SF5 and IM62 (i.e. Parental carpel-biased). We performed GO term enrichment analysis using the PlantRegMap online server (http://plantregmap.cbi.pku.edu.cn/index.php). IM62 = *M. guttatus* IM62 parent, SF5 = *M. nasutus* SF5 parent, FER = fertile RSB_7 introgression hybrid, STE = sterile RSB_7 introgression hybrid, Annotated = count of the 20431 genes expressed in our dataset with GO term ID, Observed = count of genes in gene set with GO term ID, Expected = count of genes in gene set with GO term ID expected if there was no enrichment, *p*-value = uncorrected *p*-value, *q*-value = corrected *p*-value

Table S5. Regulatory category gene counts. Table reports counts and percentages of heterozygous introgression genes that fall into different regulatory divergence categories across FER and STE carpels and stamens (see Figure 7). Also listed are the counts of genes exhibiting parental expression divergence (SF5-IM62 divergent), genes parental expression conservation (SF5-IM62 similar), and total expressed genes (Total) in each genomic region across the different genotype-tissue groups. Percentages of *cis* only,

trans only, *cis* + *trans* reinforcing, *cis* + *trans* opposing, and *cis* x *trans* are calculated based on the number of genes exhibiting parental expression divergence (SF5-IM62 divergent), whereas percentages of Compensatory, Conserved and Ambiguous are based on genes exhibiting parental expression conservation (SF5-IM62 similar). IM62 = *M. guttatus* IM62 parent, SF5 = *M. nasutus* SF5 parent, FER = fertile RSB_7 introgression hybrid, STE = sterile RSB_7 introgression hybrid, RSB = FER or STE RSB_7 introgression hybrid

Table S6. Relationship between gene expression and regulatory divergence in STE stamens. Contingency table shows distribution of heterozygous introgression genes that are misexpressed or not (Other) in STE stamens across three regulatory divergence super-categories: (i) Compensating *cis* and *trans* (i.e. *cis* + *trans* opposing, *cis* x *trans*, or compensatory), (ii) Conserved, and (iii) Other (i.e. *cis* only, trans only, *cis* + *trans* reinforcing, or Ambiguous). STE = sterile RSB_7 introgression hybrid

Table S7. Relative gene expression at *hms1* and *hms2*. Table summarizes results from pairwise comparisons of differential gene expression (DGE tests) across the eight genotype-tissue groups in this study for the 16 genes in the mapped regions of *hms1* and *hms2*. DGE tests were conducted using the glmTreat function in edgeR (Robinson *et al*., 2010). Relative transcript abundance (log2 fold-change) is shown for significant (log2 fold-change > 1.25, FDR ≤ 0.05) DGE tests. * = FDR ≤ 0.05, ** = FDR ≤ 0.005, *** = FDR ≤ 0.001, n.s. = not significant, n.t. = not tested (excluded from analysis because transcript abundance was below one count-per-million (CPM)), IM62 = *M. guttatus* IM62 parent, SF5 = *M. nasutus* SF5 parent, FER = fertile RSB_7 introgression hybrid, STE = sterile RSB_7 introgression hybrid, st = stamen, cp = carpel

Table S8. Data availability. Raw sequencing reads were deposited to the Sequence Read Archive (SRA) database under BioProject PRJNA604758, entitled "RNAseq of stamens and carpels from Mimulus guttatus IM62, M. nasutus SF5, and sterile (STE) and fertile (FER) SF5-IM62 introgression hybrids."
