# Supplementary figures and images for: Rampant Misexpression in a Mimulus (Monkeyflower) Introgression Line Caused by Hybrid Sterility, Not Regulatory Divergence
Source: Mol Biol Evol. 2020 Mar 20;37(7):2084–98. doi: 10.1093/molbev/msaa071 (PMC7306685; doi:10.1093/molbev/msaa071)

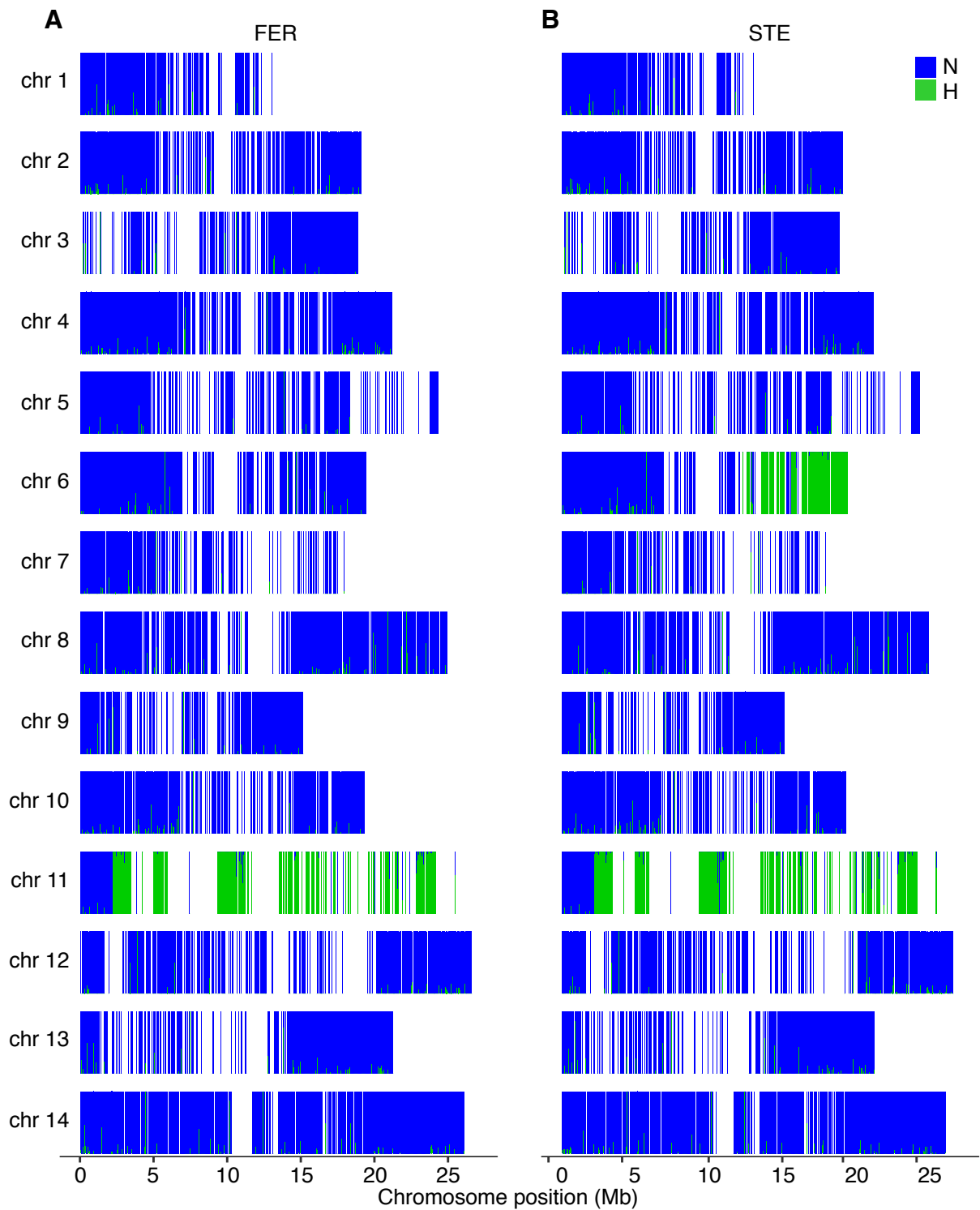

Supplement: msaa071_Supplementary_Data [file msaa071_supplementary_data.zip › msaa071-Suppl_Data/Figure S1 MBEresub - RSB genotype plot 50kb bins.pdf]

**A**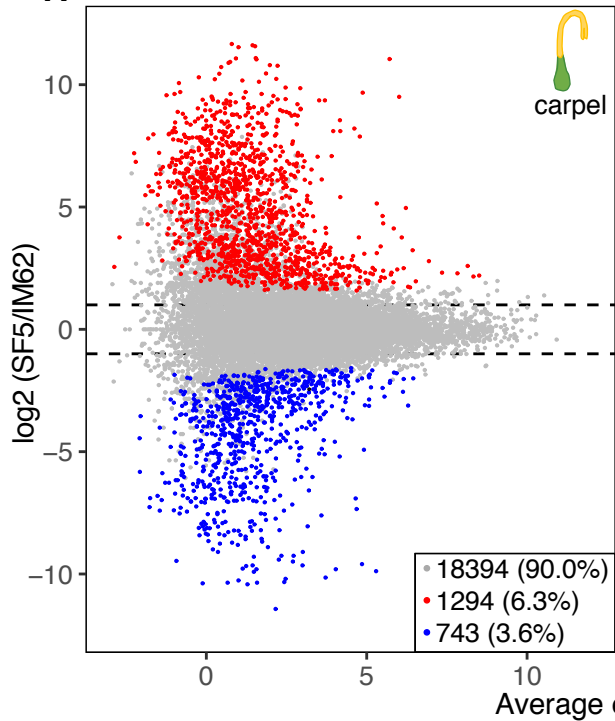**B**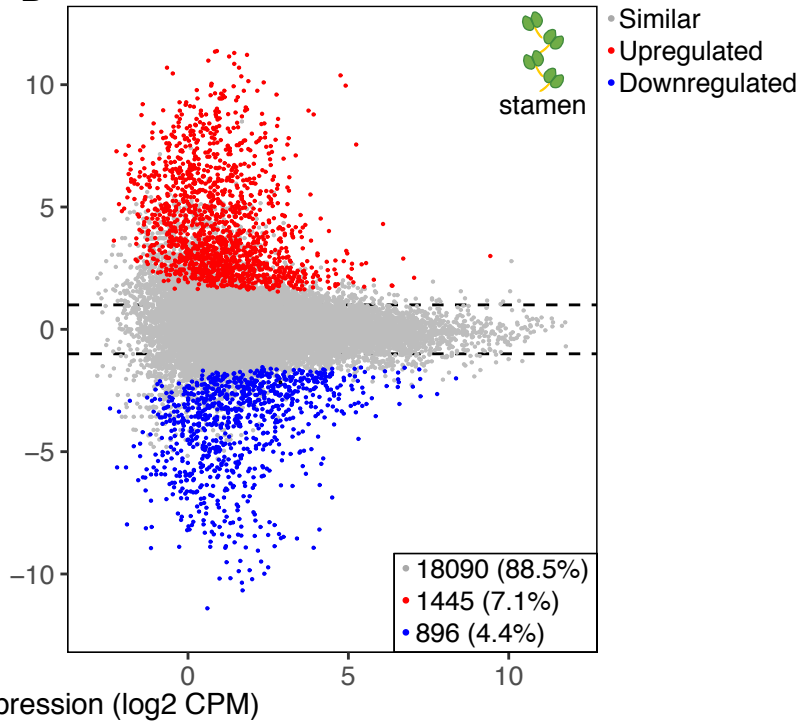

Supplement: msaa071_Supplementary_Data [file msaa071_supplementary_data.zip › msaa071-Suppl_Data/Figure S2 MBEresub - SF5 vs IM62 Expression.pdf]

Relative transcript abundance (log<sub>2</sub> fold-change)

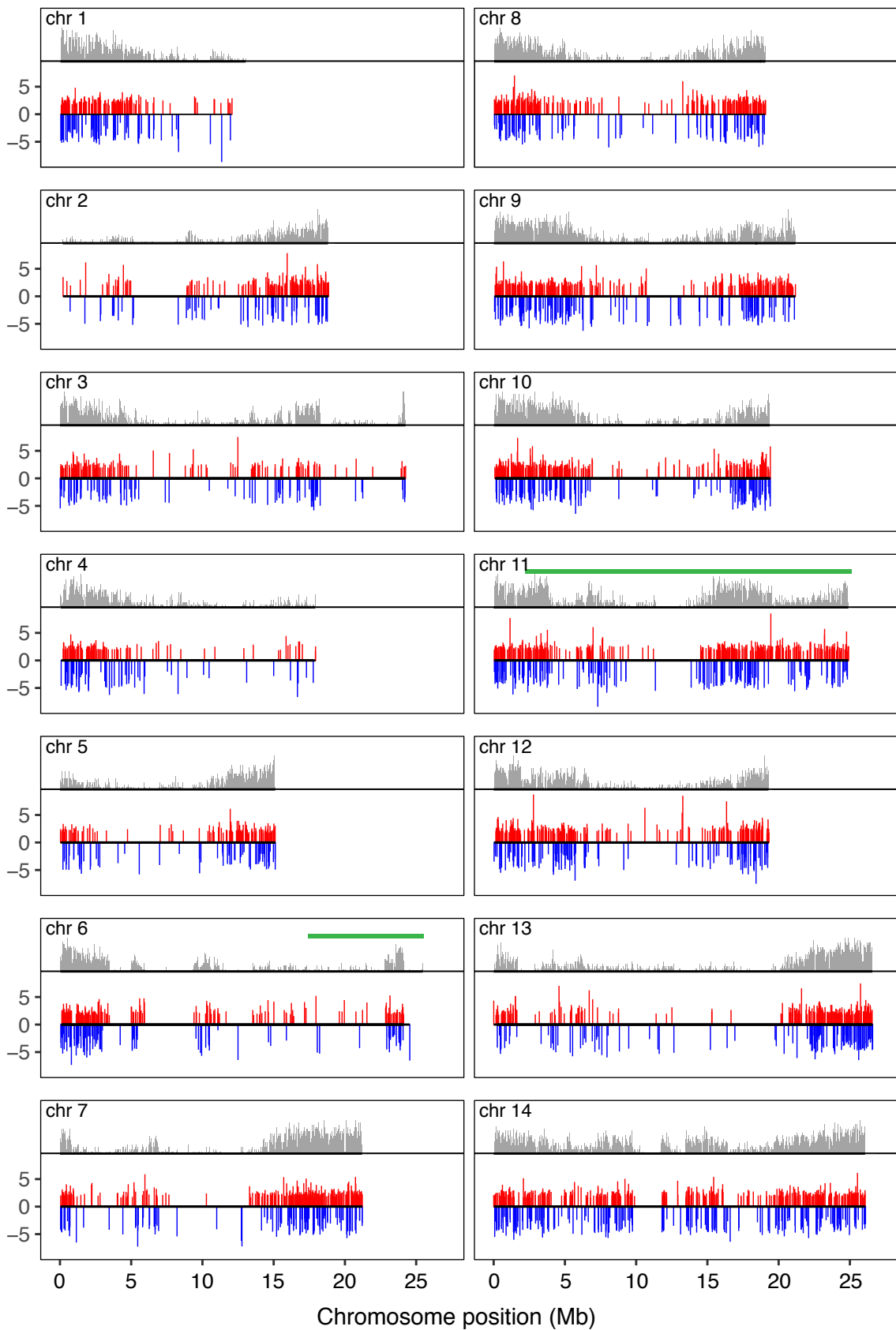

Supplement: msaa071_Supplementary_Data [file msaa071_supplementary_data.zip › msaa071-Suppl_Data/Figure S3 MBEresub - STEst DEG distribution plot.pdf]

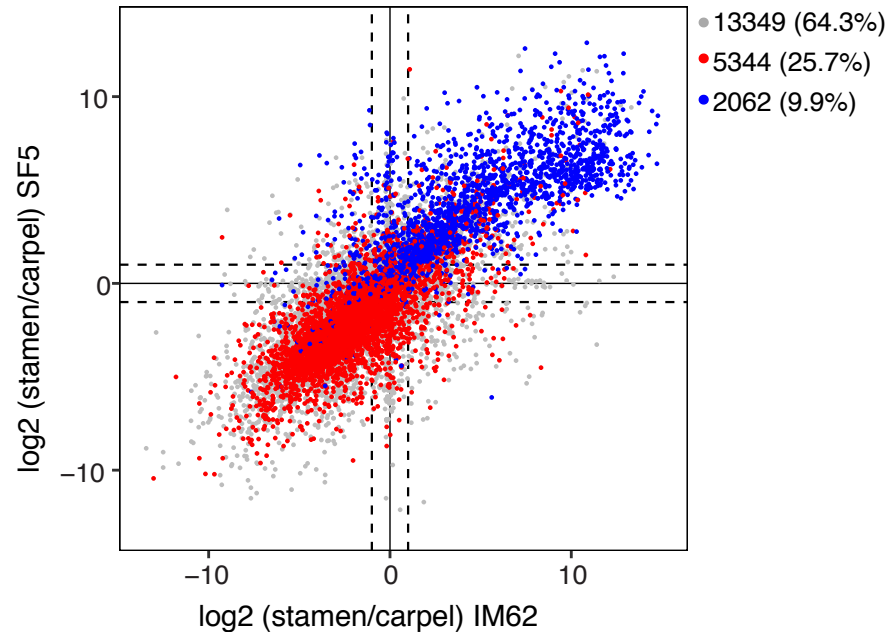

Supplement: msaa071_Supplementary_Data [file msaa071_supplementary_data.zip › msaa071-Suppl_Data/Figure S4 MBEresub - STEst expression of tissue-biased genes.pdf]

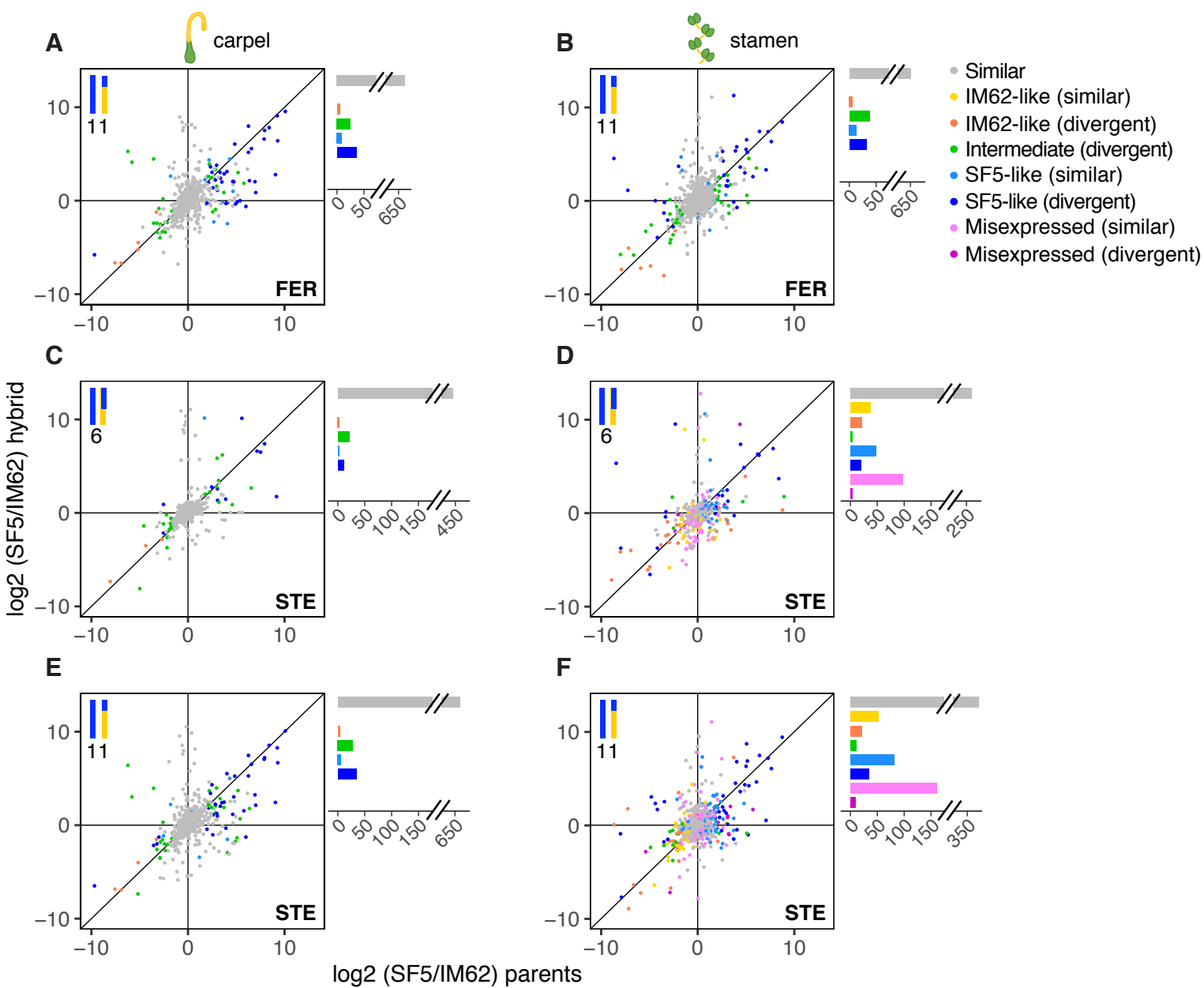

Supplement: msaa071_Supplementary_Data [file msaa071_supplementary_data.zip › msaa071-Suppl_Data/Figure S5 MBEresub - FER & STE Exp cat pFC vs aFC scatter Jan2020.pdf]

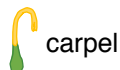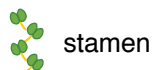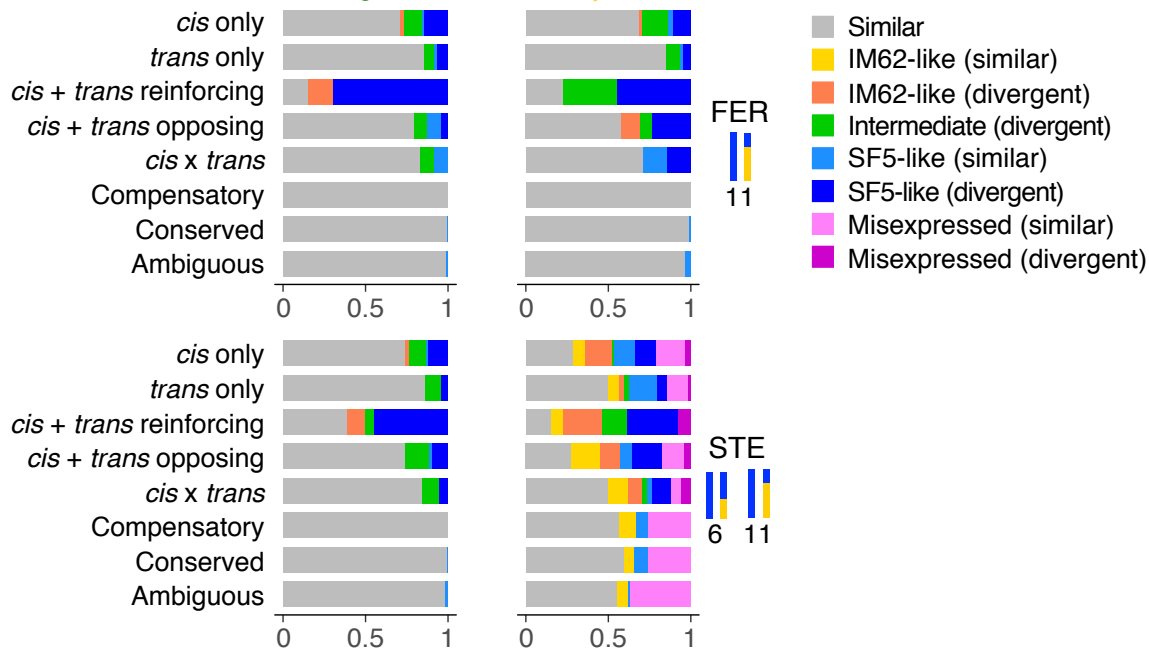

Supplement: msaa071_Supplementary_Data [file msaa071_supplementary_data.zip › msaa071-Suppl_Data/Figure S6 MBEresub - Prop Exp.Categories in Reg.categories Jan2020.pdf]
